# A scoping review of digital interventions for the promotion of mental health and prevention of mental health conditions for young people

**DOI:** 10.1093/oodh/oqaf005

**Published:** 2025-02-02

**Authors:** Evangelia Baka, Yi-Roe Tan, Brian Li Han Wong, Zhongyue Xing, Peiling Yap

**Affiliations:** HealthAI, Rue de Varembé 7, 1202, Geneva, Switzerland; Information Service Science, Faculty of Economics and Management, University of Geneva, 1205, Geneva, Switzerland; HealthAI, Rue de Varembé 7, 1202, Geneva, Switzerland; Department of International Health, Care and Public Health Research Institute, Maastricht University, Universiteitssingel 40, 6229 ER, Maastricht, Netherlands; Digital Health Section, European Public Health Association (EUPHA), Otterstraat 118, 3513 CR, Utrecht, The Netherlands; Digital Public Health Task Force, Association of Schools of Public Health in the European Region (ASPHER), UM Brussels Campus, Av de Tervueren 153, BE-1150, Brussels, Belgium; School of Public Health and Preventive Medicine, Faculty of Medicine, Dentistry and Health Sciences, The University of Melbourne, 161 Barry St, Carlton VIC 3010, Australia; HealthAI, Rue de Varembé 7, 1202, Geneva, Switzerland

**Keywords:** digital health, digital mental health, digital technologies, mental health, prevention, promotion, young people

## Abstract

Digital mental health (DMH) interventions leveraging digital technologies, such as mobile applications, web-based platforms, artificial intelligence and wearable devices, have emerged as a promising avenue for addressing the mental health needs of young people. This scoping review examines the landscape of primary and secondary preventive DMH interventions for young people aged 10–24 years. Six electronic databases were searched, leading to a final incorporation of 81 studies published between 2010 and 2022. Each of these studies corresponds to a unique DMH intervention. Our findings reveal that research activity in the area of promotive and preventive DMH interventions started gaining ground from 2019 onwards, with the majority of studies conducted in Australia and the USA. 70% of the total studies targeted the prevention of mental health conditions. Randomized controlled trials were the predominant study methodology, while mental well-being, depressive disorders, anxiety disorders, life skills and disorders specifically associated with stress were the most targeted mental health or well-being conditions. Finally, mobile applications and web interfaces were the most studied form of DMH interventions. Most of these applications have integrated advanced AI/ML algorithms to serve the purpose of personalization and real-time monitoring. However, there is a marked need for more emphasis on preventive and, especially, promotive mental health measures, as well as the active inclusion of low- and middle-income countries in future research.

## INTRODUCTION

The onset of any mental disorder or disruption of well-being often occurs in childhood and adolescence, with 75% of mental health (MH) problems being established before the age of 25 and 50% of them by the age of 14 [1,2]. This highlights the importance of early interventions, which support the promotion and maintenance of well-being, as well as the prevention and detection of MH pre-clinical risk factors and early signs, for the growth and development of young people. MH interventions exist in a spectrum, consisting of promotion, prevention, early intervention, treatment, recovery and maintenance [3]. In this review, we focused on digital mental health (DMH) interventions for the promotion of MH and prevention of MH conditions for young people. Preventive interventions aim to mitigate the development of MH disorders by addressing the risk factors and early signs of MH conditions, while promotive interventions focus on enhancing well-being and resilience. In general, the promotion of MH involves strategies such as fostering healthy relationships and coping mechanisms, while prevention targets the reduction of incidence and even the symptom severity of MH conditions. In this review, we focused on primary and secondary preventive interventions to address early signs and risk factors before the onset of clinical MH disorders. Tertiary prevention, which involve managing symptoms, restoring health or providing rehabilitation for individuals with existing diagnosis, is therefore not included in this review.

In this digital era, DMH interventions leveraging digital technologies, like mobile applications, web-based platforms, artificial intelligence (AI) and wearable devices, offer promising solutions for the MH needs of young people. These interventions can overcome traditional barriers such as accessibility, affordability and stigma associated with face-to-face interventions. Recent perspectives, such as those outlined by The Lancet & Financial Times Commission on Governing Health Futures 2030, emphasize the concept of ‘digital childhoods’ and underscore the importance of improving DMH services as a means of prioritizing children and young people in a digitally transformed universal healthcare system [4]. Young people increasingly view digital health technologies as a crucial tool to address their MH concerns, as detailed in reports like the Digital Health Futures Report which provides insights into young people’s use and opinions of digital health technologies for MH improvement [5]. Furthermore, a recent analysis on optimizing adolescent well-being in a digital age highlights the potential of digital healthcare to enable health professionals to reach adolescents in remote and underserved communities, as it opens avenues for adolescents to self-manage and monitor their physical and mental health [6].

In light of these developments, it is imperative to have a comprehensive understanding of the landscape of digital interventions focused on MH promotion and/or the prevention of MH conditions among young people. Globally, numerous reviews have attempted to identify DMH interventions, exploring their design, efficacy/effectiveness and ethical challenges in addressing the growing burden of MH disorders. Interventions have been reviewed across the age span of young people, covering MH disorders like anxiety [7–10], depression [8–11], suicide [12,13], eating disorders [14] or social complex needs [15] and examining specific types of interventions like video games [16], as well as online [2,14], and mobile applications [15,17,18]. However, the majority of existing reviews concentrate on late prevention, early intervention and treatment. Further upstream interventions such as promotion of wellbeing and prevention of MH disorders is much less explored despite its potential to reduce the overall prevalence and severity of MH conditions before they require more intensive treatment. The accessibility of these upstream interventions can depend on technology used and country of development. Interventions using more contemporary technology such as AI may offer enhanced adaptability and predictive capabilities, but their success can be contingent on the technological infrastructure and resource availability, which often vary significantly between low- and middle-income countries (LMICs) and high-income countries, impacting overall intervention outcomes [19].

This scoping review aims to fill the above-mentioned gap by having a comprehensive understanding of the current research and development (R&D) landscape of promotive and preventive DMH interventions for young people by identifying, mapping and reporting on the full breadth of evidence in this domain. Scoping reviews offer a broad overview of existing literature, identifying key concepts and gaps in research, especially in complex or emerging fields, without requiring quality assessment of studies. Their primary aim is to inform future research directions and provide a synthesized summary of available evidence, making it a valuable tool in the preliminary stages of evidence synthesis [20]. Specifically, we identified the current R&D efforts on DMH interventions, noting gaps and emerging technologies. We also extracted information on how the DMH interventions were validated and benchmarked. Lastly, we discussed how one can use the learnings in LMIC settings.

## MATERIALS AND METHODS

Prior to conducting the scoping review, a detailed protocol outlining the objectives, research methods and anticipated results was established, but not registered (supplementary material). We used a 5-stage methodological framework, first proposed by Arksey and O’Malley, consisting of the following steps: (i) Definition of the research questions; (ii) Identification of similar reviews to validate our idea; (iii) Selection of relevant studies that will be examined; (iv) Screening and filtering of the data, consisting of abstract screening, full-text screening and full-text extraction; (v) Descriptive analysis and report writing [21]. PRISMA-ScR checklist was used for the reporting, as presented in the supplementary materials [22].

### Eligibility criteria

Guided by our research question of ‘what is the current global state of promotive and preventive DMH interventions for young people?’, we defined our inclusion criteria using the Population/Concept/Context (PCC) framework. Studies included contained:

- **Population:** Healthy young people with a mean age between 10 and 24 years, as defined by the World Health Organization [23];
- **Concept:** Promotive and preventive DMH interventions;
- **Context:** Global, with a timeframe from the inception of each database to August 2022;
- **Article type:** All types of peer-reviewed publications, except for abstracts only, conference proceedings, opinion pieces and reviews;
- **Language:** English

Studies were excluded if they contained one or more of the following criteria:

- Treatment/curative interventions;
- Pure teleconferencing and/or routine clinical data collection (electronic health records);
- Neurodevelopmental disorders and physical deformities;
- Participants with pre-existing mental or other conditions;
- Interventions targeting only parents/caregivers;
- Desired age range not explicitly stated.

For studies referring to the same intervention, only one study per DMH intervention was included. The study included is based on the following order: randomized controlled trials (RCTs); Studies with research findings; Studies without findings (such as protocol papers, technology design description). The rationale is to extract as much informative data as possible, without duplicating counts of each DMH intervention.

### Search strategy

The search was conducted in six electronic databases in August 2022: PubMed, Ovid MEDLINE, Cochrane Database of Systematic Reviews (Reviews and Trials), Scopus, PsycNet (PsycArticles, PsycInfo) and Google Scholar.

In order to come up with our final search string, we experimented with various search queries as indicated in Table 1. Based on the initial search numbers, we concluded on this final search string: (Child*[tiab] OR Youth[tiab] OR ‘Young people’[tiab] OR Teenag*[tiab] OR Adolescen*[tiab] OR ‘School’[tiab]) AND (‘Mental health’[tiab] OR ‘wellbeing’[tiab] OR ‘well-being’[tiab] OR ‘mental well-being’[tiab] OR psychiatr*[tiab] OR psychol*[tiab] OR ‘mental disorder’[tiab]) AND (‘Digital health’[tiab] OR ‘digital medicine’[tiab] OR ‘ehealth’[tiab] OR ‘e-health’[tiab] OR ‘mhealth’[tiab] OR ‘m-health’[tiab] OR ‘virtual health’[tiab] OR ‘telehealth’[tiab] OR ‘telemedicine’[tiab] OR ‘artificial intelligence’ [tiab] OR ‘AI’[tiab]). Prevent*[tiab] was not included because it narrowed the scope substantially, as seen from string S5 in Table 1, and some of the retrieved articles cover both prevention and treatment. We considered this aspect during the screening stage instead. Details of search strings used in each of the databases can be found in Supplementary Table 1.

**Table 1:** The buildup of the PubMed search query.

**String**	**Query**	**Results (*n*)**
S1	Child*[tiab] OR ‘Youth’[tiab] OR ‘Young people’[tiab] OR Teenag*[tiab] OR Adolescen*[tiab] OR ‘School’[tiab]	1 957 270
S2	‘Mental health’[tiab] OR ‘wellbeing’[tiab] OR ‘well-being’[tiab] OR ‘mental well-being’[tiab] OR psychiatr*[tiab] OR psychol*[tiab] OR ‘mental disorder’[tiab]	22
S3	Prevent*[tiab]	1 661 460
S4	‘Digital health’[tiab] OR ‘digital medicine’[tiab] OR ‘ehealth’[tiab] OR ‘e-health’[tiab] OR ‘mhealth’[tiab] OR ‘m-health’[tiab] OR ‘virtual health’[tiab] OR ‘telehealth’[tiab] OR ‘telemedicine’[tiab] OR ‘artificial intelligence’[tiab] OR ‘AI’[tiab]	526 367
S5	(Child*[tiab] OR Youth[tiab] OR ‘Young people’[tiab] OR Teenag*[tiab] OR Adolescen*[tiab] OR ‘School’[tiab]) AND (‘Mental health’[tiab] OR ‘wellbeing’[tiab] OR ‘well-being’[tiab] OR ‘mental well-being’[tiab] OR psychiatr*[tiab] OR psychol*[tiab] OR ‘mental disorder’[tiab]) AND (Prevent*[tiab]) AND (‘Digital health’[tiab] OR ‘digital medicine’[tiab] OR ‘ehealth’[tiab] OR ‘e-health’[tiab] OR ‘mhealth’[tiab] OR ‘m-health’[tiab] OR ‘virtual health’[tiab] OR ‘telehealth’[tiab] OR ‘telemedicine’[tiab] OR ‘artificial intelligence’[tiab] OR ‘AI’[tiab)	249
Final	(Child*[tiab] OR Youth[tiab] OR ‘Young people’[tiab] OR Teenag*[tiab] OR Adolescen*[tiab] OR ‘School’[tiab]) AND (‘Mental health’[tiab] OR ‘wellbeing’[tiab] OR ‘well-being’[tiab] OR ‘mental well-being’[tiab] OR psychiatr*[tiab] OR psychol*[tiab] OR ‘mental disorder’[tiab]) AND (‘Digital health’[tiab] OR ‘digital medicine’[tiab] OR ‘ehealth’[tiab] OR ‘e-health’[tiab] OR ‘mhealth’[tiab] OR ‘m-health’[tiab] OR ‘virtual health’[tiab] OR ‘telehealth’[tiab] OR ‘telemedicine’[tiab] OR ‘artificial intelligence’[tiab] OR ‘AI’[tiab])	1272

### Screening and data extraction

To ensure the quality of our screening and extraction processes, each title, abstract and full text were screened by two independent reviewers using the Covidence software, and inter-rater reliability was computed using Cohen’s Kappa (supplementary material). Subsequently, for each included study, two reviewers extracted data independently. A data extraction form that is relevant for the review objective and questions was created and tested by the study team before use. Any disagreements that arose between the reviewers at any stage were resolved through discussion among the study team to maintain consistency in decision-making.

The following data items were extracted: [1] study title, author(s), aim of the study, country where the study was conducted and year of publication; [2] intervention name, intervention characteristics, including the targeted element of the MH intervention spectrum (promotion and/or prevention), population description, the targeted MH/well-being condition, the setting and the duration; [3] methodology, including study design, number of participants, inclusion and exclusion criteria; and [4] quantitative and qualitative outcome measures.

### Data analysis

All extracted data were collated into a summary table (Supplementary Table 2), which provided an overview of the key characteristics of each study. After familiarization with the data, the data from each study was mapped and categorized into pre-defined domains: timeline, study design, geographic location, targeted MH/well-being conditions and intervention type (targeted MH intervention spectrum element, and intervention technology type) to enable a clearer understanding of the evolving landscape and to better analyze the gaps in the existing literature. For each domain, we conducted a descriptive numerical summary to quantify the characteristics of the included studies. In addition, a narrative summary was used to present the results of each domain to describe the key points, patterns, trends and gaps from the literature reviewed. Tables, charts and figures were also created whenever relevant to summarize the data in a clear and concise manner.

Classification of the timeline, study design and geographic location were done through simple extraction of the information in the text of the studies. The targeted MH/well-being conditions were classified using a combination of standardized classification system (WHO ICD-11) for MH disorders [23] and commonly used terms for mental well-being. Each DMH intervention can target more than one condition, resulting in the total count being larger than the number of included studies. Interventions were classified as promotion and/or prevention by two independent reviewers of the study team to minimize subjective bias. A standard definition of promotion and prevention interventions was predefined to ensure alignment. Our approach to the classification of technology type aimed to differentiate traditional DMH interventions such as text messages, mobile applications and web interfaces from emerging DMH interventions such as AI/Machine learning (ML), wearable devices and virtual reality (VR). The categories of technology and interventions originate from existing publications. Interventions can be classified as more than one technology type if applicable. This reflects the reality where DMH interventions typically manifest in more than one format.

## RESULTS

**Figure 1:**
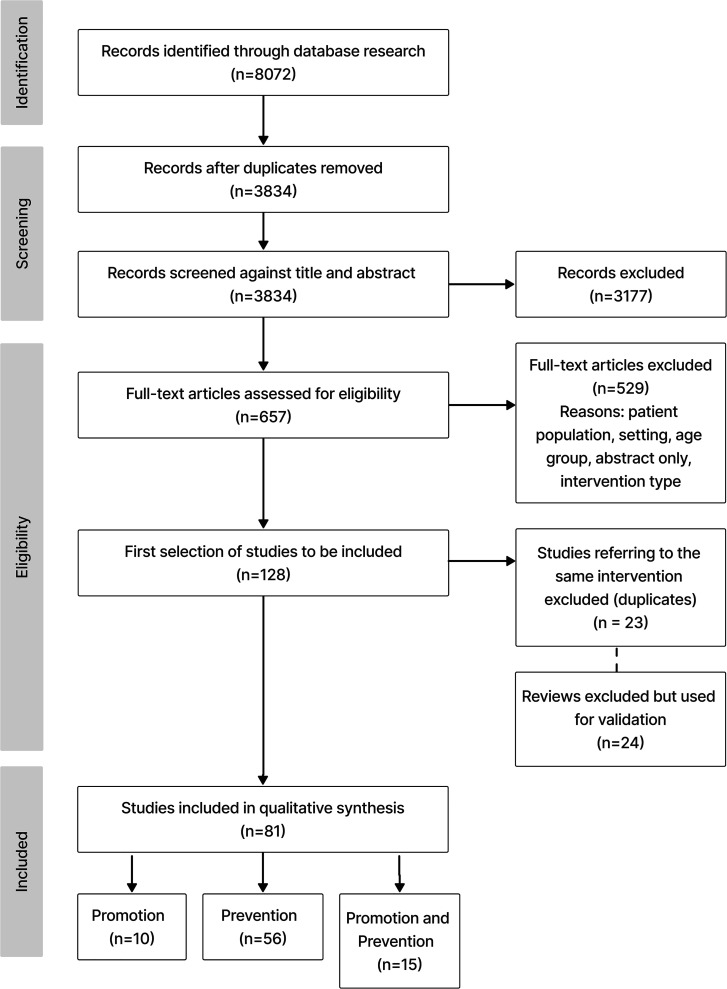
PRISMA flow diagram of the selection process.

The initial search yielded 3834 studies. After the abstract screening process, 657 full text articles were assessed for relevance according to the defined eligibility criteria, resulting in the final inclusion of 81 articles for this scoping review. Each of these articles corresponds to a unique DMH intervention. The study selection procedure is described in Fig. 1.

### Timeline

The extracted 81 studies were published between 2010 and 2022. Few studies were published before 2017, with one study published in 2010 [24], two in 2014 [25,26] and one in 2015 [27]. A slight publication increase was observed for 2017 (n = 5) [28–32] and 2018 (n = 7) [33–39]. Research activity gained ground in 2019 (n = 12) [40–51] and 2020 (n = 15) [52–66] followed by a peak in publications in 2021 (n = 24) [67–90]. However, a slight decrease in the number of published studies was noted for 2022 (n = 14) [91–104] which can be justified as our review included publications until August 2022.

### Study designs

Majority of the studies (43%) were RCTs [24–26,28,30,33–35,38,40–42,44,45,47,48,52,54,56–59,61,63,66,71,75–77,80,95,99–101,103], followed by 20% cohort studies [27,36,39,43,46,50,64,70,72,73,78,88–91,102], and 11% AI/ML training/validation studies [32,37,49,51,60,65,86,98,104]. The remaining studies were qualitative research [53,55,62,79,81,84,97], descriptive studies [31,87], non-randomized experimental trials [82,92], cross-sectional studies [93,94], technology design and development [29,67,74,83,85], case series [68], case–control [96] and economic evaluations [69]. The objectives of these studies could be divided into four main categories: 40% tested the efficacy/effectiveness of the interventions, 15% evaluated the feasibility, acceptability and usability of the interventions, 17% looked at both the efficacy/effectiveness and feasibility of the interventions, and another 25% described the design and development of the interventions. The remaining small percentage looked at economic evaluations. Among the RCTs, almost 30% of them are protocol papers without reported findings [38,40,41,47,57,59,75–77,103]. Of those with outcomes reported, symptoms related to psychological distress (48%) [25,28,42,44,48,57,59,75–77,80,100], depression (44%) [25,28,41,42,47,52,57–59,76,77] and anxiety (40%) [25,28,35,41,52,57–59,76,77] were the most frequently measured primary health outcomes, with depression and anxiety almost always measured together by tools such as Depression Anxiety Stress Scale – short form [105]. Mental well-being (48%) [25,30,45,48,52,58,59,61,71,75,76,80] and health-related quality of life (32%) [26,38,40,45,61,75,76,95] were frequently measured as secondary health outcomes using tools such as adaptations of the Warwick-Edinburgh mental well-being scale [106] and World Health Organization Quality of Life Instrument-Brief [107].

### Geographic location

The included studies were predominantly conducted in Australia (n = 19) [25,30,38,41,46,48,52,54,59,64,66,69,76,77,79,84,85,94,96] and the USA (n = 17) [15,28,35,42–44,48,50,60,66,68,70,71,87,88,90,93], followed by the United Kingdom (n = 6) [45,61,62,73,86,97] and New Zealand (n = 6) [31,34,36,65,72,83]. The remaining studies were conducted in Canada (n = 4) [33,63,82,99], the Netherlands (n = 4) [24,26,75,102], India (n = 3) [29,37,49], Indonesia (n = 2) [74,103], Switzerland (n = 2) [32,80], Norway (n = 1) [104], Taiwan (n = 1) [100], Russia (n = 1) [67], Romania (n = 1) [101], Qatar (n = 1) [98], Portugal (n = 1) [39], the Philippines (n = 1) [89], South Korea (n = 1) [92], Kenya (n = 1) [47], Japan (n = 1) [95], Ireland (n = 1) [56], Hong Kong (n = 1) [78], Germany (n = 1) [40], Finland (n = 1) [51], Colombia (n = 1) [53], Chile (n = 1) [55], Brazil (n = 1) [58] and Afghanistan (n = 1) [27]. Out of the 81 DMH interventions presented in this scoping review, 11 of them were developed in LMICs, accounting for only 14% of contributions [27,29,37,47,49,53,58,67,74,89,103]. In terms of study settings, the majority of studies were conducted in a community setting (52%) [24,25,27,29–32,34,35,37,41–43,46,49,51–53,58,59,64–66,71,72,75,77–79,81–86,88,91,93,94,99,102–104] or a school-based environment (41%) [26,28,33,36,38–40,44,45,48,50,54–57,61–63,67–69,73,74,76,80,89,90,92,95–98]. The remaining 7% of the studies were conducted in a clinical setting such as children’s clinics, virtual clinics and antenatal care units for young mothers [47,60,70,87] or an adjustable environment such as intervention delivered in VR devices or web-based gaming platforms [100,101].

**Figure 2:**
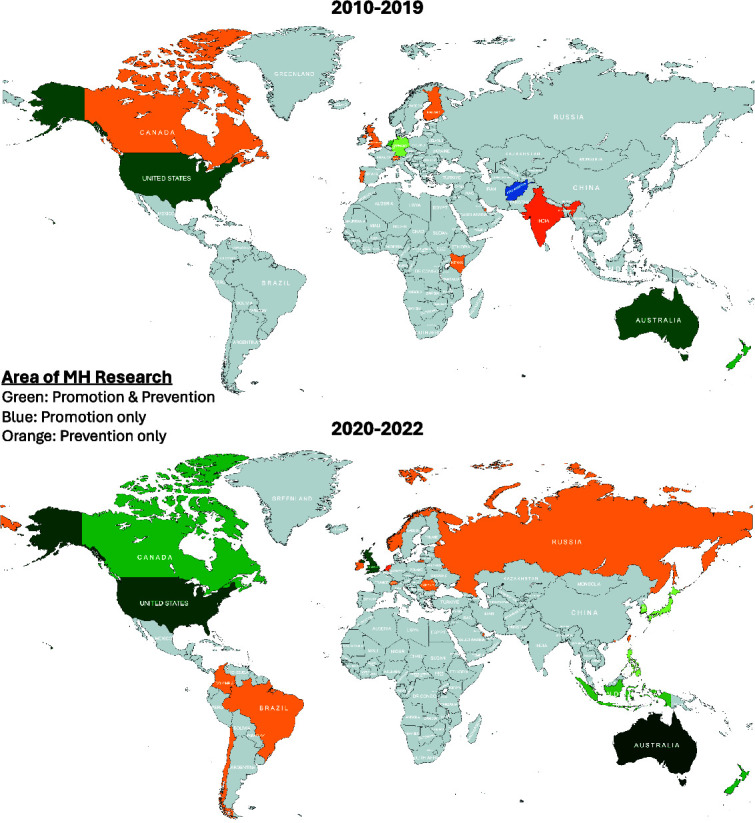
R&D of preventive and promotive DMH interventions conducted and published according to countries across two time periods (2010–2019; 2020–2022). The colors represent the area of MH research conducted and published in each country. The gradient represents the number of studies from the largest (darkest) to the smallest (lightest).

Figure 2 further illustrates the R&D progress of preventive and promotive DMH interventions conducted and published (in English) by all countries across two time periods, namely 2010–2019 and 2020–2022. The colors indicate the area of MH research conducted and published by each country, namely the promotion of mental health, prevention of mental health conditions or research looking at both promotion and prevention. In 2014, publications in Australia started and remained a preeminent research force in the domain of preventive and promotive DMH interventions. Conversely, countries such as Afghanistan, India and Kenya, having already a very low number of studies, exhibited no further research or a possible inadequately documented trajectory of research activities since 2020. Research on preventive DMH interventions was first published in Australia and the USA in 2014 and 2017 respectively and witnessed an exponential expansion spanning the years 2020–2022. Brazil, Chile, Colombia, Hong Kong, Ireland, Norway, Russia, Romania and Taiwan also started publishing research on preventive DMH interventions from 2020 onwards. Finally, research publications pertaining to promotive DMH interventions remained notably little throughout the two time periods, with New Zealand and Australia contributing to more than half of it. In 2020, research publications on promotive DMH interventions expanded to Canada, Indonesia, Japan, South Korea, the United Kingdom and the Philippines.

### Targeted mental health and well-being conditions

**Figure 3:**
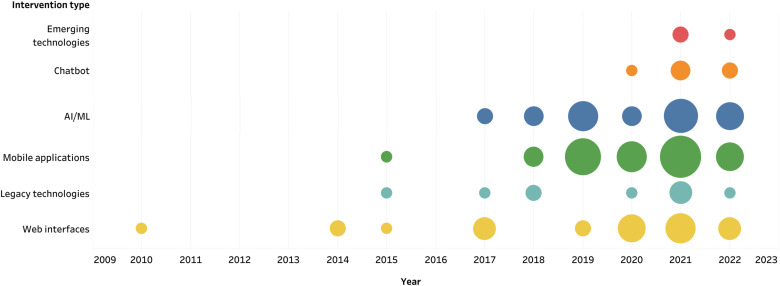
Mental health and well-being conditions addressed by the extracted interventions sorted by the frequency of their appearance.

Each of these DMH interventions targeted various MH or well-being conditions, independently or combined. The majority addressed mental well-being (18%) [24–26,30,34,36,37,39,45,52–54,56,58,59,63,65,67,72,73,78,79,81–83,85,89,90,93,94,97,102], depressive disorders (16%) [25,27,28,31,33,34,38,41,43,46,47,52,55,57,59,64,72,76,77,91,92,96,102,104] anxiety disorders (15%) [25,28,31,33–35,38,41,43,46,50,52,59,64,69,72,76,77,86,91,96,102], life skills (13%) [24,28,34,45,48,50,61,63,65,66,77,80,81,83,90,92,93,99,101] and disorders specifically associated with stress, including trauma (10%) [33,39,41,44,48,56,72,81,82,85,87,93,100]. Mental well-being encompasses emotional stability, well-being awareness, psychological resilience and social connectedness. Life skills include help-seeking, coping skills, gratitude and life satisfaction. Intentional self-harm (e.g. suicide) [31,59,66,68,92], disorders due to substance use or addictive bahaviors [27,38,54,74,76,80,91], emotion regulation [29,48,50,73,77,94,98] and self-esteem/self-awareness [34,56,62,67,75,103] have also been targeted by some interventions. Figure 3 portrays the comprehensive MH and well-being conditions that have been addressed by the extracted interventions.

### Intervention type

The interventions were categorized based on the targeted MH intervention spectrum element, namely promotion and prevention, as well as the technology used. The majority of interventions targeted prevention (n = 57) [24,28,29,31–33,35,37–39,41,42,44–51,53–61,63,64,67–81,86,87,90,91,93,96–100,102,104], corresponding to 70% of the total number of interventions listed in this review, whereas a few of them (n = 10) are related to the promotion of MH [26,27,30,34,36,62,65,66,83,94]. There is also a number of interventions targeting both promotion and prevention (n = 14) [25,40,43,52,63,76,82,84,85,88,89,92,95,103], like MindGuide [24] which looked at the general promotion of MH and the prevention of depression or WeClick [52] which aims to improve social self-efficacy and belongingness as well as to prevent depressive and anxiety symptoms.

**Figure 4:**
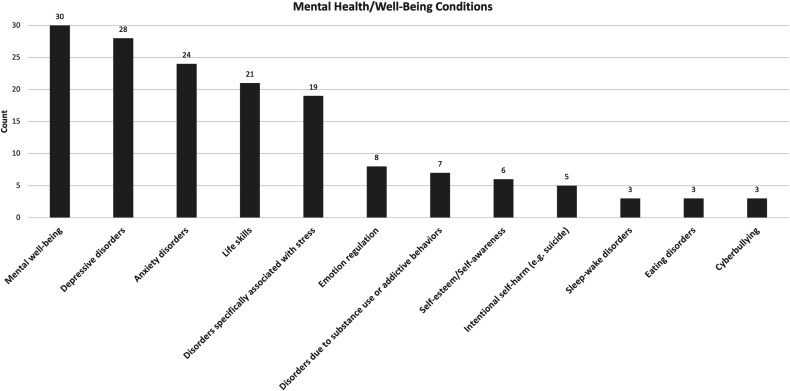
The evolution of the intervention types over the last decade from inception until 2022. The colors indicate the type of technology used for the interventions while the sizes represent the number of studies associated with each intervention type per year.

The technology categories behind each intervention included: Mobile applications (34%) [33,36,39,42–50,52,56,57,59,61–64,67,70–73,76,79–81,83,88,90,94,95,98,102,103], AI/ML (26%) [15,31,32,35,37,39,46–52,59,60,64,67,68,78–83,87,98,101–104], Web interfaces (23%) [24–26,28–31,40,53–55,61–63,69,74,76,78,82,90,92,93,99,101], Foundational technologies, including videos, online questionnaires and text messages (9%) [27,32,34,38,58,74,75,77,96,97], Chatbot (5%) [65,84,85,89,100] and Emerging technologies, including wearable devices and the use of VR (3%) [86,87,100]. Although AI/ML technology is also deployed in some of the above technologies, we chose to include a separate AI/ML category (simply referred to as ‘AI/ML’ hereafter) which consists of algorithmic descriptions or very technical interventions that do not have a specific application yet. Figure 4 presents the evolution of the intervention types over time. DMH interventions predominantly manifest in a binary format, encompassing both web-based platforms and smartphone applications [61–63,76] or the latter and AI attributes [39,46–50,52,59,64,67,79–81,83,98,102,103]. Additionally, some interventions exhibit a composite nature, comprising paired components such as video courses along with mobile or web applications [27,74,75]. However, there are DMH interventions consisting of only foundational technologies, such as video, online questionnaires and SMS. Moreover, most of the contemporary DMH interventions, which predominantly manifest as mobile or web applications, have integrated advanced AI/ML algorithms to serve the purpose of personalization and real-time monitoring. On the other hand, there exist instances of purely AI/ML systems like the SPACEs platform [60] which is designed to identify and analyze social determinants and adverse childhood experiences from information sources, the Sensus app [35] which is devised for the collection of GPS data to prognosticate social anxiety, and the application of AI techniques for the detection of cyberbullying [51]. Chatbots have also been used, especially in community settings [65,84,85,100]. Lastly, the use of emerging technologies has recently gained ground with the noteworthy examples of Shaukat-Jali et al, who tested a wearable device to detect social anxiety [86], Schaefer et al. who used VR environments for biofeedback-assisted relaxation [87], and Trappey et al. who combined a chatbot with VR to create an empathy-centric counseling system [100]. In addition, the integration of gaming and art into several interventions started in 2018. Grow it [102], REThink [101], Match Emoji [83] and MorALERT [99] are gaming applications, while Music eScape [48], BYOTS [50] and MoodyTunes [94] are music-based applications and lastly, ESRA [98] is an AI platform translating children’s drawings.

### Studies conducted in LMICs

With a focus on research conducted in LMICs, Table 2 provides a comprehensive overview of the 11 published studies along with their respective attributes, namely study design, study aims, study population characteristics, targeted MH/well-being condition and intervention [27,29,37,47,49,53,58,67,74,89,103]. In Asia, seven studies were conducted and published in India, Indonesia, Afghanistan and the Philippines. The studies targeted a variety of conditions, such as addiction, self-esteem, body image and psychological distress, through mainly preventive DMH interventions. The interventions were predominantly delivered through mobile applications, with some having AI/ML integrated. In South America, two studies were conducted and published in Brazil and Colombia, while Kenya was the only country in Africa that published on DMH interventions for young people. Both the studies in Brazil and Kenya targeted pregnant women to ensure well-being and prevent perinatal depression. In Colombia, co-design workshops with young adults and health professionals were conducted to culturally adapt a web-based Mental Health eClinic for young people. These were followed by testing and evaluation sessions. Finally in Russia, a digital health passport was developed for monitoring physical and mental health in relation to the education progress of youths. A similar overview for all the other included studies can be found in Supplementary Table 2.

**Table 2:** An overview of the 11 studies conducted and published in LMICs.

**Author (year)**	**Country (setting)**	**Study design**	**Study aim**	**Sample characteristics**	**Targeted MH condition (MH spectrum)**	**Intervention**
Aziz et al. (2021)	Indonesia (schools)	Technology design & development	To explain and describe the use of expert system to diagnose online game addiction to junior high school students.	**Age (years):** 12–16 **Population type:** Students **Sample size:** 1000	Gaming addiction (Prevention)	**Name:** - **Type:** Foundational technology; web interface **Description:** Four steps for developing this system 1. designing architecture of expert system, 2. representing knowledge, 3. designing database, 4. testing and implementing the system. Data input in the database includes user, addiction, symptom, question, solution and rule data.
Fatori et al. (2020)	Brazil (community)	RCT	To test the efficacy of an intervention on maternal parenting and well-being and to investigate the compliance rate.	**Age (years):** 14–19 **Population type:** Pregnant women in an urban deprived area **Sample size:** 25	Well-being (Prevention)	**Name:** Primeiros Laços **Type:** Foundational technology **Description:** Home-visiting intervention delivered by trained nurses targeting 1. health and social care, 2. environmental health, 3. life course, 4. parenting skills, 5. family and social support.
Garbett et al. (2022)	Indonesia (community)	RCT	To evaluate the effectiveness of Warna-Warni Waktu, which aims to reduce state and trait body dissatisfaction and improve mood among young Indonesian women.	**Age (years):** 15–19 **Population type:** Young women **Sample size:** 1800	Body image; self-esteem; mood disorders (Promotion and prevention)	**Name:** Warna-Warni Waktu **Type:** Mobile application; AI/ML **Description:** Six 5-minute videos, with each video supplemented with up to five brief interactive activities. The activities encourage the target audience to reflect and apply the learnings from the videos to their own lives.
Gowda et al. (2019)	India (community)	AI/ML training/validation	To assess the mood state spectrum of a person over time and validate the same by correlating with salivary cortisol, psychologist assessment results.	**Age (years):** Not mentioned **Population type:** Young people **Sample size:** 21	Mood disorders (Prevention)	**Name:** - **Type:** Mobile application; AI/ML **Description:** Model is deployed through an application. The application monitors the mood of the user in real time through camera. Mood state spectrum is generated through the data, which will be used by physicians in early detection and treatment of any mood disorders.
Green et al. (2019)	Kenya (hospital)	RCT	To test the Healthy Moms intervention with pregnant women and new mothers recruited from public hospitals.	**Age (years):** ≥18 **Population type:** Pregnant women and new mothers **Sample size:** Not mentioned	Perinatal depression (Prevention)	**Name:** Healthy Moms **Type:** Mobile application; AI/ML **Description:** Uses an existing AI system called Tess, deployed through a mobile phone to drive automatic conversations with users by employing active listening techniques. Participants will be prompted to rate their mood via a short message service, and track and reflect on their mood and behaviors on a daily basis using the physical Healthy Moms journal.
Gillis G. (2015)	Afghanistan (community)	Cohort	To design and test an intervention for strengthening a mental health system that improves awareness in the community, informs health practitioners and makes treatment accessible.	**Age (years):** 18–25 **Population type:** Young adults **Sample size:** 1200	Depression; drug addiction (Promotion)	**Name:** - **Type:** Foundational technology; mobile application **Description:** SMS messages are sent to young adults in the community to create awareness about MH problems. Smartphone application for health providers to empower them to provide MH services in the community. Also integrated an eLearning platform and telehealth.
Ospina-Pinillos et al. (2020)	Colombia (community)	Qualitative	To conduct co-design workshops to culturally adapt MHeC for young people in Colombia, and perform one-on-one user-testing sessions to evaluate an alpha prototype of MHeC-C.	**Age (years):** 16–30 (Median = 19.5 for co-design workshops; 22 for user testing) **Population type:** Young people **Sample size:** 18 for co-design workshops, 10 for user testing	Well-being (Prevention)	**Name:** MHeC-C **Type:** Web interface **Description:** 6 iterative phases: co-design workshops; knowledge translation; tailoring to language, culture, and place (or context); and one-on-one user-testing sessions (alpha, beta, delta prototype).
Sia et al. (2021)	Philippines (schools)	Cohort	To describe the design and evaluation of Abot to encourage students to improve their lifestyle habits and well-being.	**Age (years):** 17–18 **Population type:** Students **Sample size:** 25	Well-being (Promotion and prevention)	**Name:** Abot **Type:** Chatbot **Description:** Chatbot deployed in Facebook Messenger. 1. Assessment (healthy or unhealthy lifestyle): discusses a specific aspect of well-being or lifestyle habit through story sharing. 2. Counselling: advice and solutions in changing lifestyle habits given. 3. Evaluation: user to give feedback on the usefulness of the conversation in encouraging him/her to practice better lifestyle habits.
Srivastava et al. (2017)	India (community)	Technology design & development	To develop an electronic platform for e-psychology systems to deal with various health issues of adolescents.	**Age (years):** 10–19 **Population type:** Adolescents and young adults **Sample size:** Not mentioned	Psychological distress (Prevention)	**Name:** Yuva **Type:** Web interface **Description:** Electronic platform for e-psychology systems by using ICT tools and services and can be used to deal with various health issues of adolescents. It incorporates several facilities like self-assessment tests, registration facility for counsellors, dieticians, instructors, etc… so they can be contacted in their hour of need.
Srividya et al. (2018)	India (community)	AI/ML training/validation	To apply ML algorithms to identify the state of mental health in a target group.	**Age (years):** 18–21 **Population type:** Students **Sample size:** 656	Psychological distress; Well-being (Prevention)	**Name:** - **Type:** AI/ML **Description:** Questionnaire looks at 5 factors: engagement, perseverance, optimism, connectedness and happiness. Based on scores obtained, a person’s MH status is predicted as mentally distressed, barely satisfied with life and optimistic.
Vorobiev et al. (2021)	Russia (schools)	Technology design & development	To describe a digital health passport for monitoring physical, mental health and physical preparedness of the educational process for participants in modern educational environment.	**Age (years):** 6–17 **Population type:** Students **Sample size:** Not mentioned	Well-being (Prevention)	**Name:** Health Passport **Type:** Mobile application; AI/ML **Description:** Objective evaluation of mental health through psychological methods such as The Ladder Test and The Self-Esteem Test. The program can also store statistically relevant information on individual and group performance; conduct comparative analysis; and provide personalized practical recommendations by analyzing test results.

## DISCUSSION

This scoping review aimed to comprehensively investigate the existing evidence base surrounding promotive and preventive DMH interventions for young people. The ubiquity of digital technology and its multiple modalities facilitates a wide variety of intervention types, each with distinct strengths and limitations. This review monitors the progress in R&D in this domain, reflecting a substantial surge in recent years with a peak of publications observed in 2021. It is plausible that factors such as the COVID-19 pandemic and the rapid proliferation of digitalization catalyzed the need to generate more evidence on the use of digital technologies to meet evolving mental health needs.

A significant proportion of the included studies were RCTs, indicating a strong interest in establishing a robust evidence base for the efficacy of DMH interventions. Despite this predominance, a substantial number of studies employed different methodologies, including cohort studies and AI/ML validation studies. As we shift towards applying more AI/ML in DMH interventions, it is essential that participatory designs as well as best ethical practices are embedded in the study methodology to minimize bias and improve robustness, explainability, and wider applicability of these interventions [4,79,108]. In this review, we noted that only eight out of the 81 studies included participatory and co-design approaches. Furthermore, the lack of pragmatic, hybrid intervention trials that assess real-world effectiveness, feasibility and acceptability of DMH interventions highlights one of the areas which future research can focus on.

Several gaps were identified in the current literature. A lack of clarity around the duration of interventions and the specifics of follow-up procedures in many studies impedes a thorough understanding of long-term outcomes and sustainability. Moreover, 70% of the DMH interventions in this review targeted the prevention of MH conditions and research pertaining to the promotion of MH only started emerging from year 2020. This imbalance between promotive and preventive actions verifies the need to move further upstream towards the promotion of MH, with community-oriented interventions as well as individualized actions. Considering that today’s youth are extensively engaged with digital technologies, it is crucial to focus more on conditions arising from increased social media use, such as sleep difficulties, cyberbullying, eating disorders, issues with self-awareness and intentional self-harm, which represented less than a quarter of the literature reviewed [104]. This gap indicates a need for these topics to be more prominently featured in global R&D efforts. Addressing these issues is essential to understanding and improving the MH of the digital-native generation.

This review has also revealed a geographic gap, with an overrepresentation of studies from high-income countries (HICs) and an insufficient focus on LMICs. This gap could stem from studies either remaining unpublished (grey literature) or published in a local language, which is beyond the scope of this review. The notable volume of published DMH studies from the USA, Australia, New Zealand and the United Kingdom, especially in MH promotion, is also worth mentioning and may be attributed to several factors such as the geographical expanse of these countries, the country’s healthcare priorities, MH prevalence, technological advancement level, research funding availability and cultural emphasis on MH within public health policies. The Australian government, e.g. has incorporated DMH services into its official website, providing public access to information on the significance of these services and guidance on how to utilize them [109].

The low number of published studies on DMH interventions in LMICs highlights the need for more R&D efforts, and their dissemination, to support evidence-based creation, adaptation, implementation and scaling of DMH interventions in the promotion of mental well-being and prevention of MH conditions for young people in these countries. Given that the type of MH conditions and suitable DMH interventions are different in HICs and LMICs due to factors such as differing social determinants of health, future studies can focus on tailoring relevant DMH interventions to address the unique challenges faced in LMICs.

In terms of technology utilized, this review has demonstrated a significant range in the types of DMH interventions, spanning from mobile applications to AI-driven platforms. This heterogeneity indicates flexibility in designing interventions that can be adapted to the needs and preferences of the targeted population. Moreover, the recent emergence of AI/ML-driven interventions highlights a growing interest in leveraging emerging digital technologies for MH solutions.

## STRENGTHS AND LIMITATIONS

To the best of our knowledge, no other scoping review has presented such a global overview of promotive and preventive DMH interventions for young people. Although our approach has led to the inclusion of a larger pool of studies, compared to other similar scoping reviews, it has enabled a more thorough representation, comparison and assessment of existing DMH interventions. Such extensive coverage highlights the areas where further R&D efforts are necessary to enhance promotive and preventive DMH strategies, ultimately aiming to improve the MH and well-being of young people.

Despite the valuable insights of this scoping review, we acknowledge the existence of several limitations. Firstly, the review may be constrained by publication bias, particularly given the overrepresentation of studies from HICs and scarcity of publications from LMICs. Language barriers might have also limited the inclusion of relevant studies published in non-English languages. Moreover, no MeSH terms were included in the search string, which could have identified additional relevant records. Lastly, ethical considerations, privacy concerns, co-design and participatory methodologies, barriers to effective use of DMH interventions by young people, the effectiveness of DMH interventions and their impact on the health outcomes of young people present ongoing challenges that might not be fully addressed within the scope of this review. Future work can further investigate these areas to have a comprehensive understanding of the potential of DMH interventions in addressing the MH needs of young people.

## CONCLUSION

This review underscores the considerable growth and potential of DMH interventions in addressing MH and well-being issues among young populations. The recent proliferation of research publications in this domain indicates increased recognition of the important potential role of digital technologies in promoting youth MH globally. The breadth of research designs and technologies used denote the extensive opportunities for future R&D.

However, this review also illuminates the current shortcomings in the field. Future R&D efforts should focus on designing sustainable interventions with a long-term perspective, broadening the scope to include more emphasis on promotive and preventive MH measures. Publication and dissemination of R&D efforts should also be strengthened to encompass diverse geographical settings, particularly LMICs. Such an approach will ensure a global and comprehensive outlook towards youth MH interventions. By focusing future R&D efforts in the identified areas, we can fully leverage the potential of digital technologies to promote mental well-being and prevent MH disorders amongst youth, ultimately fostering healthier future societies for generations to come.

## Supplementary Material

Supplementary_Materials_(combined)_oqaf005

## Data Availability

All data generated and/or analyzed during this study are included in this published article (and supplementary materials).
