# Supplementary material for: A scoping review of digital interventions for the promotion of mental health and prevention of mental health conditions for young people
Source: Oxf Open Digit Health. 2025 Feb 2;3:oqaf005. doi: 10.1093/oodh/oqaf005 (PMC11932149; doi:10.1093/oodh/oqaf005)
Supplement: Supplementary_Materials_(combined)_oqaf005 [file Supplementary_Materials_(combined)_oqaf005.pdf]

Supplementary Materials

A Scoping Review of Digital Interventions for the Promotion of Mental Health and Prevention of Mental Health Conditions for Young People

Evangelia Baka[+1,2], Yi-Roe Tan[+1], Brian Li Han Wong*[3,4,5], Zhongyue Xing[6], Peiling Yap[1]

[1] HealthAI, Geneva, Switzerland
[2] Information Service Science, Faculty of Economics and Management, University of Geneva, Geneva, Switzerland
[3] Department of International Health, Care and Public Health Research Institute, Maastricht University, Maastricht, The Netherlands
[4] Digital Health Section, European Public Health Association (EUPHA), Utrecht, The Netherlands
[5] Digital Public Health Task Force, Association of Schools of Public Health in the European Region (ASPHER), Brussels, Belgium
[6] School of Public Health and Preventive Medicine, Faculty of Medicine, Dentistry and Health Sciences, The University of Melbourne, Melbourne, Australia

[+]Co-authors: These two authors contributed equally to the work reported here.

*Correspondence: Brian Li Han Wong (brian.wong@maastrichtuniversity.nl)
Universiteitssingel 40, 6229 ER Maastricht, Netherlands

<u>ORCiD numbers:</u>
Evangelia Baka: 0009-0006-0498-5996
Yi-Roe Tan: 0000-0002-4013-7330
Brian Li Han Wong: 0000-0001-8709-5847
Zhongyue Xing: 0000-0002-5119-973X
Peiling Yap: 0000-0002-5964-8210

eTable 1. Final search strings used in PubMed, Ovid MEDLINE, Cochrane Database of Systematic Reviews (Reviews and Trials), Scopus, PsycNet (PsycArticles, PsycInfo), and Google Scholar.

**PubMed**	**Ovid MEDLINE**	**Cochrane Database of Systematic Reviews (Reviews and Trials)**	**Scopus**	**PsycNet (PsycArticles, PsycInfo)**	**Google Scholar**
(Child*[tiab] OR Youth[tiab] OR "Young people"[tiab] OR Teenag*[tiab] OR Adolescen*[tiab] OR “School”[tiab]) AND (“Mental health”[tiab] OR “wellbeing”[tiab] OR “well-being”[tiab] OR “mental well-being”[tiab] OR psychiatr*[tiab] OR psychol*[tiab] OR “mental disorder”[tiab]) AND (“Digital health”[tiab] OR “digital medicine”[tiab] OR “ehealth”[tiab] OR “e-health”[tiab] OR “mhealth”[tiab] OR “m-health”[tiab] OR “virtual health”[tiab] OR “telehealth”[tiab] OR “telemedicine”[tiab] OR “artificial intelligence”[tiab] OR “AI”[tiab])	(Child*.tw. OR Youth.tw. OR "Young people".tw. OR Teenag*.tw. OR Adolescen*.tw. OR School.tw.) AND ("Mental health".tw. OR wellbeing.tw. OR well-being.tw. OR "mental well-being".tw. OR psychiatr*.tw. OR psychol*.tw. OR "mental disorder".tw.) AND ("Digital health".tw. OR "digital medicine".tw. OR ehealth.tw. OR e-health.tw. OR mhealth.tw. OR m-health.tw. OR "virtual health".tw. OR telehealth.tw. OR telemedicine.tw. OR "artificial intelligence".tw. OR AI.tw.)	(Child*:ti,ab OR Youth:ti,ab OR "Young people":ti,ab OR Teenag*:ti,ab OR Adolescen*:ti,ab OR School:ti,ab) AND ("Mental health":ti,ab OR wellbeing:ti,ab OR well-being:ti,ab OR "mental well-being":ti,ab OR psychiatr*:ti,ab OR psychol*:ti,ab OR "mental disorder":ti,ab) AND ("Digital health":ti,ab OR "digital medicine":ti,ab OR ehealth:ti,ab OR e-health:ti,ab OR mhealth:ti,ab OR m-health:ti,ab OR "virtual health":ti,ab OR telehealth:ti,ab OR telemedicine:ti,ab OR "artificial intelligence":ti,ab OR AI:ti,ab)	(TITLE-ABS(Child*) OR TITLE-ABS(Youth) OR TITLE-ABS("Young people") OR TITLE-ABS(Teenag*) OR TITLE-ABS(Adolescen*) OR TITLE-ABS(School)) AND (TITLE-ABS("Mental health") OR TITLE-ABS(wellbeing) OR TITLE-ABS(well-being) OR TITLE-ABS("mental well-being") OR TITLE-ABS(psychiatr*) OR TITLE-ABS(psychol*) OR TITLE-ABS("mental disorder")) AND (TITLE-ABS("Digital health") OR TITLE-ABS("digital medicine") OR TITLE-ABS(ehealth) OR TITLE-ABS(e-health) OR TITLE-ABS(mhealth) OR TITLE-ABS(m-health) OR TITLE-ABS("virtual health") OR TITLE-ABS(telehealth) OR TITLE-ABS(telemedicine) OR TITLE-ABS("artificial intelligence") OR TITLE-ABS(AI))	(Child*.ti,ab. OR Youth.ti,ab. OR "Young people".ti,ab. OR Teenag*.ti,ab. OR Adolescen*.ti,ab. OR School.ti,ab.) AND ("Mental health".ti,ab. OR wellbeing.ti,ab. OR well-being.ti,ab. OR "mental well-being".ti,ab. OR psychiatr*.ti,ab. OR psychol*.ti,ab. OR "mental disorder".ti,ab.) AND ("Digital health".ti,ab. OR "digital medicine".ti,ab. OR ehealth.ti,ab. OR e-health.ti,ab. OR mhealth.ti,ab. OR m-health.ti,ab. OR "virtual health".ti,ab. OR telehealth.ti,ab. OR telemedicine.ti,ab. OR "artificial intelligence".ti,ab. OR AI.ti,ab.)	Child|"Youth"|"Young people"|Teenage|Adolescent|"School" "Mental health”|wellbeing|“well-being”|“mental well-being”|psychiatr|psychol|“mental disorder” “Digital health”|“digital medicine”|“ehealth”|“e-health”|“mhealth”|“m-health”|“virtual health”|“telehealth”|“telemedicine”|“artificial intelligence”|“AI”

eTable 2. Supplementary table for all studies

**Author (year) [reference]**	**Country (setting)**	**Study design**	**Study aim**	**Sample characteristics**	**Targeted MH condition (MH spectrum)**	**Intervention**	**Outcome measures**	**Summary of findings**
Albritton et al. (2021) [68]	USA (schools)	Case series	To investigate a piloted digital mental health training program (Be Present) for youth, specifically describing the impact on youth behavioural outcomes and user engagement and identifying high-risk youth in the early phases of prevention.	**Age (years):** 14-22 **Population type:** Youth **Sample size:** 65	Suicide (Prevention)	**Name:** Be Present **Type:** AI/ML **Description:** e-learning campaign for Be Present advocates targeting youth suicide prevention.	**Quantitative:** Self-efficacy; Intentions; Behaviors; Social support; Knowledge; Self-esteem; Sources of strength. **Qualitative:** Engagement evaluation; Identification of high-risk responses.	There was a significant increase only to behaviors and social support. There was also a gradual decline in engagement, probably due to the duration of the intervention (1 year). Most respondents who received escalated high-risk response messages were female.
Ali et al. (2022) [98]	Qatar (schools)	AI/ML training/ validation	To test the effectiveness of art therapy-based emotion-sensing using a AI-based mobile application, that can help parents and teachers understand the emotions of children by analyzing their drawings.	**Age (years):** Not mentioned **Population type:** Youth/child **Sample size:** 102 (primary data); 521 (secondary data)	Emotions (Prevention)	**Name:** Emotion Sensing Recognition App (ESRA) **Type:** Mobile application; AI/ML **Description:** The ResNet50 convolutional neural network model was implemented for the classification of drawings. Drawings were classified into positive or negative emotions.	**Quantitative:** Accuracy; Sensitivity; Specificity; Precision. **Qualitative:** The particular pixels of the image that were responsible for the prediction of that image as positive or negative.	Accuracy ranged from 55% to 79% in the four experiments.Sensitivity ranged from 33% to 86% whereas specificity ranged from 46% to 83%. Precision ranged from 60% to 74%. One obvious pattern that was observed from the heatmaps was that of the detection of the yellow color as positive and the red color as negative. Bright colors, flowers, trees, and

								greenery depicted positive clues, whereas, war vehicles, weapons, and hospitals depicted negative cues.
Ammar et al. (2020) [60]	USA (hospitals)	AI/ML training/ validation	To leverage explainable AI, and propose a proof-of-concept prototype for a knowledge-driven evidence-based recommendation system to improve mental health surveillance for adverse childhood experiences.	**Age (years):** Not mentioned **Population type:** Children/adults **Sample size:** Not mentioned	Adverse childhood experiences (Prevention)	**Name:** Semantic Platform for Adverse Childhood Experiences (SPACEs) **Type:** AI/ML **Description:** A QA conversation agent is used to extract information regarding social determinants and adverse childhood experiences. When the assessment is complete, there is a real-time recommendation service.	**Quantitative:** - **Qualitative:** Risk factors based on social determinants and adverse childhood experiences.	The prototype aims to maximize knowledge about the patient without having to delve into all of the questions that are often asked in adverse childhood experiences and social determinants assessments.
Andrews et al. (2022) [96]	Australia (schools)	Case control	To evaluate the effectiveness of a universal eHealth school-based prevention program for depression and anxiety, and the moderating role of friendship network characteristics.	**Age (years):** Mean = 15.5 **Population type:** Students **Sample size:** 2539	Depression; anxiety (Prevention)	**Name:** 'Climate schools' ehealth preventive mental health program **Type:** Foundational technology **Description:** eHealth mental health course with online cartoon components delivered to students during health education classes. Included cognitive-behavioural principles and incorporates skill acquisition, psychoeducation, management of psychological symptoms,	**Quantitative:** Internalizing symptoms; Depression and anxiety; Psychological distress and mental health knowledge. **Qualitative: -**	Across all participants, there were small but significant increases in internalizing problems, depression, and anxiety 18 months post-intervention. Higher scores compared to the control group at 6 and 12-month follow-up. Symptoms were higher for participants 15-16 years old.

						cognitive symptoms, behaviour and additional skills specific to anxiety and depression.		
Arps et al. (2018) [34]	New Zealand (community)	RCT	To investigate the feasibility and efficacy of a gratitude text-messaging program for promoting adolescent mental health relative to a positive reflective control condition.	**Age (years):** 16-21 (Mean = 18.09) **Population type:** Older adolescents and young adults **Sample size:** 136	Gratitude; life satisfaction; well-being; self-esteem; depression; anxiety (Promotion)	**Name:** National youth helpline service (Youthline) **Type:** Foundational technology **Description:** Youthline provided the technological platform for sending, receiving, and tracking the text-messages. Received a daily text-message for 28 days which attempted to prompt reflection on a specific gratitude domain.	**Quantitative:** Feasibility; Effectiveness; Gratitude; Well-being; Depression. **Qualitative:** Feedback on the text-messaging approach.	The program demonstrated positive impacts on young participants, showing increases in gratitude, subjective well-being, and decreases in depressive symptoms, especially notable in those with higher initial levels of depression. However, no significant differences were found between the two groups in terms of mental health improvements. High engagement and enjoyment were reported with the text-messaging approach, although the gratitude condition had more unanswered messages. Most participants shared messages with others and would recommend the program to peers.
Aziz et al. (2021) [74]	Indonesia (schools)	Technology design & development	To explain and describe the use of expert system to diagnose online game addiction to junior high school students.	**Age (years):** 12-16 **Population type:** Students **Sample size:** 1000	Gaming addiction (Prevention)	**Name:** - **Type:** Foundational technology; web interface **Description:** Four steps for developing this system 1. designing architecture of expert system, 2.	**Quantitative:** Addiction level. **Qualitative:** -	69% amongst total samples of Junior High School Student had a low-level addiction to online game, 25% experienced medium-level addiction, and 6% were highly addicted.

						representing knowledge, 3. designing database, 4. testing and implementing the system. Data inputted to the database include user, addiction, symptom, question, solution, and rule data.		The dominant online game players were male students. The age of online game players ranged between 12 and 15 years olds.
Bannink et al. (2014) [26]	Netherlands (schools)	RCT	To evaluate the effect of E-health4Uth and E-health4Uth and consultation on well-being and health behaviours as applied by preventive youth health care in secondary schools.	**Age (years):** Mean = 15.9 **Population type:** Students **Sample size:** 1256	Well-being; health behaviours (Promotion)	**Name:** E-health4Uth **Type:** Web interface **Description:** Received web-based tailored messages developed for adolescents by the Department of Health Promotion and Health Education of the University of Maastricht, focused on their health behaviours and well-being.	**Quantitative:** Health related QoL; Safe sex; Mental health status; Drug use; Feasibility; Satisfaction. **Qualitative:** Consultations' evaluation.	The E-health4Uth intervention slightly improved health and safe sex among Dutch teens but showed mixed results when combined with consultations. Specifically, the combined approach improved mental health in at-risk adolescents but increased drug use among boys. The study supports using E-health4Uth with consultations for teens at risk of mental health issues, as this combo led to better outcomes than the digital program alone. Participants generally responded positively to the tailored messages.
Baras et al. (2018) [39]	Portugal (schools)	Cohort	To implement a mobile application that acts as a virtual tutor and is aware of the user activities, appointments and	**Age (years):** Mean = 22.8 **Population type:** Students **Sample size:** 107	Well-being; stress (Prevention)	**Name:** - **Type:** Mobile application; AI/ML **Description:** Mobile app with notifications to incentivise and motivate students to adopt healthy	**Quantitative:** General phone usage; Regularity. **Qualitative:** Mood description between morning and afternoon.	Participants typically make and receive more calls and SMS in the afternoon than in the morning. In the first session, 'fatigue' and 'vigor' were the top mood descriptors, with no

			mood levels. Additionally, to implement an IoT deployment for a smart study room with a network of intelligent objects and to provide feedback mechanisms that may contribute to improving students' well-being, developing good studying habits and reducing stress levels in the most critical period.	(formative survey); 4 (user study); 22 (deployment)		living habits, guidelines for efficient studying and time management. IoT study rooms to complement the mobile app. Deployed sensors, single board computers and the existent infrastructure will measure the physical context of the room. Also, a wall display will be deployed for feedback purposes.		significant mood differences between morning and afternoon. In the second session, 'confusion,' 'tension,' and 'vigor' were most noted.
Batterham et al. (2019) [41]	Australia (community)	RCT	To examine the efficacy of FitMindKit in reducing depression symptoms, anxiety symptoms, suicidal ideation, and disability relative to HealthWatch and compare uptake of the program via the Internet with uptake via general practices and pharmacies.	**Age (years):** ≥18 **Population type:** Adults **Sample size:** 750	Depression; anxiety; panic; social anxiety disorder; suicidal ideation (Prevention)	**Name:** FitMindKit **Type:** Web interface **Description:** 12-module psychotherapeutic transdiagnostic online intervention that delivers cognitive behavioural therapy and other techniques via a series of brief videos and self-directed exercises.	**Quantitative:** Depression; Anxiety; Panic; Social anxiety; Suicidal ideation; Program satisfaction. **Qualitative:** -	No results; protocol paper.

Beilharz et al. (2021) [84]	Australia (community)	Qualitative	To assess preliminary acceptability and feasibility via the collection of qualitative feedback from young people and parents/carers regarding the content, structure, and design of the chatbot, in accordance with an agile methodology strategy.	**Age (years):** 13-18 **Population type:** Young people **Sample size:** 17	Body image concerns; eating disorders (Promotion and prevention)	**Name:** KIT **Type:** Chatbot **Description:** A conversation decision tree was designed that offered psychoeducational information on body image and eating disorders, as well as evidence-based coping strategies. The conversation content was based on evidence-based information/interventions for eating disorders, specifically, psychoeducation, cognitive behavioral therapy, acceptance commitment therapy, and mindfulness, and adapted for delivery by a chatbot. Preliminary online forums were held with young people to enhance co-design of KIT and refine the dialog, particularly devising shorter and more conversational ways of delivering educational information, as well as the design of the decision tree.	**Quantitative:** - **Qualitative:** Feedback on chatbot character and design.	Both groups of participants were satisfied with the appearance of KIT, the character, and reported that it would appeal to a wide audience of users. Non-gender specific, and non-human design of KIT is important. Majority of young people perceive blues and greens as calming colors. Specific suggestions were made regarding the brevity and tone to increase engagement.
Berryman et al. (2018) [36]	New Zealand (schools)	Cohort	To determine the validity and feasibility of the Particip8 app for student self-	**Age (years):** 21-30 (Median = 23)	Well-being (Promotion)	**Name:** Particip8 **Type:** Mobile application **Description:** Record self-reflection on well-being daily. Participants were	**Quantitative:** Self-reflected well-being scores; Feasibility.	Negative experiences reduced daily well-being scores by about 20%, with unconstructive feedback having the largest negative

			reflected well-being data collection.	**Population type:** Students **Sample size:** 29		able to select a face emoticon scale to indicate how they felt on that particular day. Additionally, participants were also asked to select from a list provided, the experiences that they had been exposed to during that day.	**Qualitative:** Experiences described by the participants.	impact. Conversely, positive experiences increased scores by roughly the same percentage. Overall, there was a median daily compliance rate of 71%, which declined over 28 days. Well-being scores were lowest on Tuesdays and highest on Saturdays, with days off generally scoring higher than days with placements.
Bhawra et al. (2021) [82]	Canada (community)	Non-randomized experimental trial	To develop and implement a sustainable digital platform that enables real-time decision-making to mitigate climate change-related impacts on food systems and mental well-being.	**Age (years):** ≥13 **Population type:** Community members **Sample size:** Not mentioned	Climate change-related mental distress (Promotion and prevention)	**Name:** FEEDS **Type:** Web interface; AI/ML **Description:** A climate change platform, which consists of a smartphone app and a digital decision-making dashboard. The app provides citizens with valuable information to mitigate health-related risks and relays big data in real time to a digital dashboard. The custom-built app uses AI to engage and enable citizens to report on environmental hazards, changes in biodiversity or wildlife, and related food and mental health issues in their communities.	**Quantitative:** Preparedness; Adaptation behavior. **Qualitative:** Changes in the knowledge or perceptions of community members about climate change impacts; Specific effects of strategies on outcomes including food security status, food sovereignty, and solastalgia.	The FEEDS Project facilitates self-determination, governance, and data sovereignty. The digital dashboard system provides decision makers with real-time data, thereby increasing the capacity to self-govern. The participatory action research approach, combined with digital citizen science, advanced the co-creation of knowledge and multidisciplinary collaboration in the digital age.

Bidargaddi et al. (2017) [30]	Australia (community)	RCT	To assess the efficacy of the web-based application "The toolbox" which aims to improve mental health and well-being.	**Age (years):** 16-25 **Population type:** Young adults **Sample size:** 387	Well-being (Promotion)	**Name:** The toolbox **Type:** Web interface **Description:** Participants first choose the areas they want to focus on, guided by an interactive quiz and subsequently receive recommendations for particular apps to download and use based on their preferences. For each recommended app, additional information is provided, including the MARS score and reviews by both health professionals and end users on what they liked and did not like, along with costs and links to download from the app store.	**Quantitative:** Well-being (emotional, psychological, social); Ecological momentary assessments (EMAs) (mood, energy, rest, sleep). **Qualitative:** -	No significant benefit on well-being at 4 weeks compared to the control group. Participants in the control group reported a significant decline in mood, energy, rest and sleep with an increasing number of logins whereas the intervention group showed no change.
Birrell et al. (2018) [38]	Australia (schools)	RCT	To describe the Climate Schools Combined (CSC) follow-up study, which aims to determine the long-term efficacy and cost-effectiveness of the CSC prevention program for depression, anxiety, and substance use up to 7 years post intervention.	**Age (years):** 13-21 **Population type:** Students **Sample size:** 6386	Depression; anxiety; substance use (Prevention)	**Name:** - **Type:** Foundational technology **Description:** Classroom lessons focused on depression, anxiety, alcohol, and cannabis. Each lesson includes both computer-based and manualized classroom activities. The computer-based component is delivered on the web via cartoon storylines that impart information about	**Quantitative:** Cannabis and alcohol use; Mental health symptoms; Cost-effectiveness; Health-related QoL. **Qualitative:** -	No results; protocol paper.

						anxiety and depressive symptoms, alcohol, and cannabis. These lessons utilize cognitive behavioural skills and strategies to assist students in identifying and reducing problematic mental health symptoms.		
Birrell et al. (2021) [76]	Australia (schools)	RCT	A study protocol to evaluate the Mind your mate program to prevent mental health, focusing on anxiety and depression and substance use problems in adolescents.	**Age (years):** 14-15 **Population type:** Students **Sample size:** 840	Depression ; anxiety; substance use (Promotion and prevention)	**Name:** Mind your Mate **Type:** Mobile application; web interface **Description:** To facilitate discussions among peers and provide key mental health literacy information about anxiety, depression, and substance use (communication tools, educational modules, suggested activities).	**Quantitative:** Primary measures (knowledge, alcohol and other substance use, MH measures - psychological distress, depression and anxiety-, help-seeking measures); Secondary measures (positive well-being, QoL, impact of covid). **Qualitative:** -	No results; protocol paper.
Boukhechba et al. (2018) [35]	USA (community)	RCT	To examine the feasibility of leveraging non-invasive mobile sensing technology to passively assess and predict college students' social anxiety.	**Age (years):** Mean = 19.43 **Population type:** Students **Sample size:** 228	Social anxiety (Prevention)	**Name:** Sensus **Type:** AI/ML **Description:** A custom mobile app (Sensus) was installed on participants' mobile phone to collect GPS data. They integrated semantic labels of locations, such as locations	**Quantitative:** Cumulative staying at each different location; Distribution of visits over time; Entropy of locations; Frequency of	Socially anxious students tend to spend more time at home and less at leisure or food venues, exhibiting fewer varied activities and preferring secure locations. They are more likely to stay in or visit supermarkets in the evenings, as opposed

						of leisure, into their prediction models to ensure a more nuanced understanding of the behavioural patterns of socially anxious individuals.	transitions between locations; Correlation between the features above and participants' social anxiety. **Qualitative:** -	to less anxious peers who prefer social settings like friends' houses. Their range of visited locations is smaller, and they often avoid public spaces, particularly during evenings and weekends. This behavior suggests a link between social anxiety and daily activity patterns.
Champion et al. (2020) [54]	Australia (schools)	RCT	To summarize the co-design and user testing of the Health4Life school-based program.	**Age (years):** Not mentioned **Population type:** Students **Sample size:** 41	Well-being (Prevention)	**Name:** Health4Life **Type:** Web interface **Description:** A web-based cartoon intervention developed to concurrently prevent 6 key lifestyle risk factors for chronic disease among secondary school students: alcohol use, smoking, poor diet, physical inactivity, sedentary recreational screen time, and poor sleep.	**Quantitative:** Physical activity; Screen time; Fruit and vegetable consumption and alcohol or tobacco use; Sleep; Attitudes, knowledge, barriers and facilitators. **Qualitative:** -	No results; protocol paper.
Daemen et al. (2021) [75]	Netherlands (community)	RCT	To investigate the efficacy of a novel, accessible, transdiagnostic ecological momentary intervention for improving self-esteem in youth with prior exposure to childhood adversity.	**Age (years):** 12-26 **Population type:** Teens and young adults **Sample size:** 174	Self-esteem (Prevention)	**Name:** SELFIE **Type:** Mobile application; Foundational technology **Description:** The intervention is based on principles of EMIs, and a guided self-help approach using principles of cognitive-behavioural therapy, aimed at modifying cognitive bias inherent to negative self-esteem and	**Quantitative:** Self-esteem; Level of momentary; Resilience; Emotional well-being; Psychological distress; General psychopathology; Clinical symptoms;	No results; protocol paper.

						developing and practicing a new behavioural repertoire guided by therapists using modelling and shaping as additional important therapeutic techniques.	Health-related QoL; Service use; Cost; Acceptability; Fidelity; Adherence. **Qualitative:** Positive and negative schematic beliefs of self; Subjective quality of life.	
David et al. (2022) [101]	Romania (any)	RCT	To investigate the validity of in-game performance measurements or scores as indicators of the game effectiveness in building real life emotion-regulation abilities.	**Age (years):** 10-16 (Mean = 13) **Population type:** Adolescents **Sample size:** 48	Emotions (Prevention)	**Name:** REThink **Type:** Web interface; AI/ML **Description:** The main goal of the game is to lead the positive character, RETMAN, and his rational friends in their quest of helping the people on Earth against the negative character, Irrationalizer, and his irrational servants. REThink has seven levels which focus on objectives based on the REBT model.	**Quantitative:** Strengths and Difficulties Questionnaire (Child version); Early Adolescent Temperament Questionnaire; Emotion Regulation Index for Children and Adolescents; The Child and Adolescent Scale of Irrationality Functional and Dysfunctional Child Mood Scales. **Qualitative:** -	Higher game scores, especially in emotion recognition (Level 1), correlate with better youth mental health, including improved moods, conduct, and peer relationships. Level 2 scores, reflecting understanding between thoughts and feelings, relate to reduced irrational beliefs. Overall, better in-game performance links to enhanced mental well-being and emotional regulation.
Deady et al.	Australia (community)	Cohort	To qualitatively explore the utility of an existing mental	**Age (years):** 16-30 (Mean	Depression ; anxiety; well-being	**Name:** HeadGear Apprentice	**Quantitative:** Feasibility: consent rates,	Engagement (both in terms of self-report and adherence) was an issue in

(2020) [64]			health app within an apprentice population, and evaluate the usability, acceptability, feasibility and preliminary efficacy of a modified version of the app (HeadGear Apprentice), designed to reduce depressive symptoms in an apprentice working population.	= 20.77, and 21.68) **Population type:** Apprentices **Sample size:** 26 (study 1); 47 (study 2)	(Prevention)	**Type:** Mobile application; AI/ML **Description:** A smartphone application-based intervention centred on behavioural activation and mindfulness therapy. 30-days challenge in which users complete one challenge daily. These include psychoeducational videos; mindfulness exercises; value-driven activity planning, goal setting, and review; and coping skill development. Risk calculator which assessed and provided participants with personalized feedback regarding their risk for future mental health issues.	adherence, attrition; Acceptability; Depression, anxiety, well-being, and work performance scores. **Qualitative:** App quality; Feedback on app.	both studies. In Study Two, users completed approximately one-third of the app's therapeutic content, with increased usage associated with improved outcomes. High self-reported scores for acceptability and utility. At follow-up, users reported improvements in all outcomes, but overall only change in well-being reached statistical significance. Good quality ratings were similar across all aspects indicating consistent degree of quality in terms of all features of the app. Low scores for customization, participants also emphasized the importance of gamification and greater personalization within the app.
Dietvorst et al. (2022) [102]	Netherlands (community)	Cohort	To evaluate whether adolescents' well-being improved after playing the multiplayer serious game app Grow It!	**Age (years):** Mean = 16.67 (first), and 18.66 (second) **Population type:** Adolescents **Sample size:** 1282	Well-being; depression; anxiety (Prevention)	**Name:** Grow It! **Type:** Mobile application; AI/ML **Description:** Adolescents monitor their emotions and behaviours in daily life by utilizing the experience sampling method (ESM). Grow It! teaches how to cope with setbacks and promotes emotional	**Quantitative:** Affective and cognitive well-being. **Qualitative:** -	53% of the adolescents increased in their affective or cognitive well-being. Adolescents with higher risk profiles (i.e., more depressive symptoms, lower atmosphere at home, and more COVID-19 impact) improved more strongly in their well-being. Positive user evaluations

				(first); 1871 (second)		resilience by offering daily challenges based on CBT.		and app engagement were unrelated to changes in affective and cognitive well-being.
Duguid et al. (2022) [94]	Australia (community)	Cross-sectional	To evaluate the usability and appeal of a first iteration of MoodyTunes to inform subsequent phases of its development.	**Age (years):** 12-25 (Mean = 17.5) **Population type:** Young people **Sample size:** 20	Mood regulation; well-being (Promotion)	**Name:** MoodyTunes **Type:** Mobile application **Description:** mHealth app that aims to increase mental health awareness in young people and to inform young people about how music can be used as an effective tool for self-regulating mood.	**Quantitative:** Usability. **Qualitative:** Positive aspects; Negative aspects.	The app was perceived in a positive manner, and as effective and well-functioning, whereas aesthetic appeal received the lowest score. Women and younger participants displayed more positive emotions while using the app. Positive aspects: links to mental health resources; ability to share music with others. Negative aspects: need to improve the aesthetic appeal; ease of use; capacity to personalize the app.
Edbrooke-Childs et al. (2019) [45]	UK (schools)	RCT	To determine the feasibility of undertaking a cluster RCT to test the effectiveness of a smartphone app, Power Up, co-designed with young people to support patient activation and shared decision-making for mental health and to empower self-	**Age (years):** 11-18 (Mean = 14.66, and 16.88) **Population type:** Children and students **Sample size:** 142	Well-being; problem management (Prevention)	**Name:** Power Up **Type:** Mobile application **Description:** A suite of tools such as My people, My diary, My plans, My questions, My decisions, Help and Support.	**Quantitative:** Feasibility (participant recruitment and retention); Acceptability. **Qualitative:** Acceptability.	Usage data showed that there were an estimated 50 (out of 64) users of Power Up in the intervention arms. Findings from the interviews indicated that young people found Power Up to be acceptable. Out of the 142 recruited participants, 45.0% (64/142) completed follow-up measures.

			management of problems.					
Edridge et al. (2020) [61]	UK (schools)	RCT	To examine the effectiveness of an mHealth intervention (ReZone) in reducing mental health difficulties in young people.	**Age (years):** 10-15 (Mean = 10.9) **Population type:** Young people **Sample size:** 409	Behavioural difficulties (Prevention)	**Name:** ReZone **Type:** Mobile application; web interface **Description:** A suite of tools such as Stress bucket, Timeout, Chill out, Art therapy, Happy faces, Game.	**Quantitative:** Emotional and behavioral difficulties; Mental well-being; Empowerment; Health-related QoL. **Qualitative:** -	No significant differences for all outcomes.
Fatori et al. (2020) [58]	Brazil (community)	RCT	To test the efficacy of an intervention on maternal parenting and well-being and to investigate the compliance rate.	**Age (years):** 14-19 **Population type:** Pregnant women in an urban deprived area **Sample size:** 25	Well-being (Prevention)	**Name:** Primeiros Laços **Type:** Foundational technology **Description:** Home-visiting intervention delivered by trained nurses targeting 1. health and social care, 2. environmental health, 3. life course, 4. parenting skills, 5. family and social support.	**Quantitative:** Depression and anxiety symptoms; Maternal parenting, parental well-being, and time spent with the child via an eDiary. **Qualitative:** -	The effect of Primeiros Laços on parental well-being may be related to the parenting skills component of the intervention. Positive effect on parental well-being and maternal parenting.
Fell et al. (2022) [93]	USA (community)	Cross-sectional	To explore the acceptability of an 8-session virtual group mind-body resiliency intervention for teen siblings of children with ASD.	**Age (years):** 14-17 (Mean = 15) **Population type:** Teens **Sample size:** 35	Resilience; stress coping (Prevention)	**Name:** SibChat **Type:** Web interface **Description:** 8-week multi-modal group program, delivered via videoconferencing, designed to teach resiliency skills and improve stress coping abilities through mind-body techniques to elicit the relaxation response, CBT strategies, and positive psychology.	**Quantitative:** Structure and content of program. **Qualitative:** Perceptions of virtual delivery; Helpful program aspects.	The intervention had the right amount of sessions (88%), structure (74%), and duration (89%). Most participants felt comfortable during sessions (74%), found it helpful to learn mind-body exercises (74%), and that the intervention helped in coping with stress (71%). The majority of participants were satisfied with virtual

						Each week focused on a different topic and included examples relevant to the sibling experience.		delivery, reporting that it was effective and convenient. However, some teens expressed mixed perceptions: they would have preferred in-person sessions to facilitate participant interaction, but the virtual delivery was acceptable given the circumstance. Teen siblings found learning resiliency techniques and making social connections to be helpful. Participants noted that the skills learned helped with a variety of issues such as sleep, stress, worry, and anxiety.
Finlay-Jones et al. (2021) [77]	Australia (community)	RCT	To outline the rationale and protocol for a single-blind RCT, comparing group Mindful Self-Compassion to a delayed-treatment waitlist control group, for improving mental health, decreasing self-criticism and increasing self-compassion in LGBTQIA+ young adults.	**Age (years):** 18-25 **Population type:** LGBTQIA+ young adults **Sample size:** Not mentioned	Depression; anxiety; stress; emotions; self-criticism; self-compassion (Prevention)	**Name:** - **Type:** Foundational technology **Description:** 8-week group program that focuses on cultivating self-compassion and mindfulness, via videoconferencing. A combination of psychoeducation, meditation practice, group interaction and interpersonal exercises.	**Quantitative:** Symptoms (depression; anxiety; stress); Self-compassion; Self-criticism and self-reassurance; Emotion regulation difficulties. **Qualitative:** Acceptability.	No results; protocol paper.

Garbett et al. (2022) [103]	Indonesia (community)	RCT	To evaluate the effectiveness of Warna-Warni Waktu, that aims to reduce state and trait body dissatisfaction and improve mood among young Indonesian women.	**Age (years):** 15-19 **Population type:** Young women **Sample size:** 1800	Body image; self-esteem; mood disorders (Promotion and prevention)	**Name:** Warna-Warni Waktu **Type:** Mobile application; AI/ML **Description:** Six 5-minute videos, with each video supplemented with up to five brief interactive activities. The activities encourage the target audience to reflect and apply the learnings from the videos to their own lives.	**Quantitative:** Trait body dissatisfaction: BESAA; Internalization of appearance ideals; Trait mood; Skin shade satisfaction; State measures of body satisfaction and mood before and after watching each video; Intervention adherence and acceptability. **Qualitative:** -	No results; protocol paper.
Gil et al. (2022) [92]	Korea (schools)	Non-randomized experimental trial	To examine the feasibility, acceptability, and preliminary outcomes of a new online self-help intervention, MindGuide, among Korean college students' families.	**Age (years):** Mean = 21.53 **Population type:** Students **Sample size:** 31	Depression; suicide; life satisfaction (Promotion and prevention)	**Name:** MindGuide **Type:** Web interface **Description:** An online self-help intervention that focuses on promoting mental health and preventing depression in college students' families. The program provides psychoeducation and activities through an illustrated narrative with CBT techniques for challenging and restructuring cognitions; mindfulness for helping clients learn how to accept and detach from thoughts	**Quantitative:** Center for Epidemiological Studies Depression Scale; Attitude Toward Suicide scale; Satisfaction With Life Scale. **Qualitative:** Perceived outcomes; Acceptability.	Participants in the risk group experienced medium to large reductions in depression. However, participants in the normal group perceived no change. Improved adaptive attitudes toward suicide prevention with medium to large within-group effect sizes. While the father in the risk group and the mother in the normal group reported a slight improvement in life satisfaction, the father in the normal group, the mother in the risk group,

						and feelings; and emotional regulation techniques for labelling, reappraisal, and expressing emotions.		and the child experienced no change. Most participants thought MindGuide helped them reduce their depressed mood by learning how to be aware of and express their feelings. They reported increased calmness and relaxation by practicing mindfulness. Some participants stated that the program made them more positive, so they were able to look at life more positively. Most participants were satisfied with the online delivery method of the program.
Glover et al. (2019) [43]	USA (community)	Cohort	To establish the feasibility and acceptability of delivering automated mental health resources via smartphone technology.	**Age (years):** 16-25 (Mean = 20.03) **Population type:** Homeless youth **Sample size:** 100	Trauma; depression; anxiety (Promotion and prevention)	**Name:** - **Type:** Mobile application **Description:** Participants received a smartphone preloaded with several apps designed to promote mental health wellness and provide real-time resources, including Pocket Helper 2.0; Koko; Illinois Warm Line; Crisis Text Line; Pocket Helper 2.0 Support System; IntelliCare Apps; and StreetLight Chicago.	**Quantitative:** Feasibility (retention); Acceptability and satisfaction; Features of app. **Qualitative:** -	Participant retention at the midpoint was moderate, with 48% of youth responding to the 3-month surveys. At 6 months, only 19% of the total sample responded to the end point survey. Overall, 63% to 68% of respondents at both time points reported benefiting from the intervention; however, participant usage and satisfaction varied with the different features. Participants reported receiving the most benefit

								from the daily tips and daily surveys. Daily tips that were most preferred by participants involved motivational tips related to overcoming struggles and making progress in life. Aside from the tips and surveys, the most used features were the app providing up-to-date resources and the automated self-help system. Interactive features, including the telephone hotline and crowd-based emotional support tool, were the least used features and were rated as the least beneficial.
Goodyear-Smith et al. (2017) [31]	New Zealand (community)	Descriptive	To outline the development, utilisation and ongoing evaluation and implementation strategies for YouthCHAT.	**Age (years):** Not mentioned **Population type:** Youth **Sample size:** Not mentioned	Depression; anxiety; suicidal ideation; substance use (Prevention)	**Name:** YouthCHAT **Type:** Web interface; AI/ML **Description:** YouthCHAT is a rapid, electronic, self-report screening tool that assesses risky health-related behaviours and mental health concerns, with a 'help question' that enables youth to prioritise areas they want help with.	**Quantitative:** - **Qualitative:** -	Description of the development of YouthCHAT.
Gowda et al. (2019) [49]	India (community)	AI/ML training/ validation	To assess the mood state spectrum of a person over time	**Age (years):** Not mentioned	Depression; anxiety (Prevention)	**Name:** - **Type:** Mobile application; AI/ML	**Quantitative:** Accuracy. **Qualitative:** -	With the inception v3 trained for 1,00,000 times with the data set that's close to 12,000 images

			and validate the same by correlating with salivary cortisol, psychologist assessment results.	**Population type:** Young people **Sample size:** 21		**Description:** Model is deployed through an application. The application monitors the mood of the user in real time through camera. Mood state spectrum is generated through the data, which will be used by physicians in early detection and treatment of any mood disorders.		classified according to the nine emotion classes as specified by psychologist, the model was able to obtain 78.4% of testing accuracy while with the near perfect training accuracy.
Green et al. (2019) [47]	Kenya (hospital)	RCT	To test the Healthy Moms intervention with pregnant women and new mothers recruited from public hospitals.	**Age (years):** ≥18 **Population type:** Pregnant women and new mothers **Sample size:** Not mentioned	Perinatal depression (Prevention)	**Name:** Healthy Moms **Type:** Mobile application; AI/ML **Description:** Uses an existing AI system called Tess, deployed through a mobile phone to drive automatic conversations with users by employing active listening techniques. Participants will be prompted to rate their mood via short message service, and track and reflect on their mood and behaviors on a daily basis using the physical Healthy Moms journal.	**Quantitative:** Depression severity; Current mood; Engagement with intervention; Feasibility; Acceptability. **Qualitative:** Barriers to access and use; Feasibility issues.	No results; protocol paper.
Grové et al. (2021) [85]	Australia (community)	Technology design & development	To describe how the Chatbot has been developed, highlighting its participatory, co-design process with	**Age (years):** 15-17 **Population type:** Young people	Stress; well-being; mindfulness (Promotion	**Name:** - **Type:** Chatbot **Description:** An element of the Chatbot is powered by AI and rules based AI using NLP. It is created to	**Quantitative:** Platform usage. **Qualitative:** Perception of chatbot; Chatbot purpose; Chatbot	Participants predominantly use Facebook, Reddit, Instagram, Snapchat applications and YouTube, Netflix, news websites. Participants used the

			youth who are the key stakeholders to benefit from this digital tool.	**Sample size:** 40 (questionnaire); 15 (interview)	and prevention)	communicate evidence based resources, wellbeing support, educational mental health information and adaptive coping strategies. The chatbot also provided links to online recommended resources.	functions and features.	internet mainly for social reading and connection. Participants found the chatbots to be interesting and exciting to engage with initially however over time they can become “boring and repetitive". The Chatbot provides students with psycho-educational and wellbeing support to encourage positive mental health. The chatbot should be used alongside a wellbeing coordinator, school psychologist, or student services team responsible for student behavior and wellbeing. The topics covered by the chatbot include both positive and negative: school, family, friends or relationships, the future, religion, books, games, art, sports, exercise and music. Youth suggested additional topics such as information about drugs/alcohol, sexuality, and identity.
Han et al. (2020) [59]	Australia (community)	RCT	To describe the protocol for a RCT to evaluate the efficacy of the LifeBuoy app for reducing suicidal	**Age (years):** 18-25 **Population type:** Young adults	Depression; anxiety; psychological distress; suicide; well-being	**Name:** LifeBuoy **Type:** Mobile application; AI/ML **Description:** Seven structured therapeutic sessions informed by	**Quantitative:** Suicidal thoughts measured by SIDAS; Suicidal behavior, depression,	No results; protocol paper.

			thoughts and behaviours, depression, anxiety, and psychological distress, and improving general mental well-being in young adults.	**Sample size:** 378	(Prevention)	dialectical behaviour therapy and incorporates the principles of positive psychology. It includes sessions on value identification, goal setting, psychoeducation, emotion regulation, and distress tolerance.	anxiety, psychological distress, and general mental well-being; Changes in the levels of insomnia, rumination, suicide cognitions, distress tolerance, loneliness, and help seeking; Satisfaction with the app; Treatment adherence. **Qualitative:** User experience.	
Haug et al. (2021) [80]	Switzerland (schools)	RCT	To test the appropriateness and short-term efficacy of a mobile phone-based life-skills training program to prevent substance use among adolescents.	**Age (years):** 14-17 (Mean = 15.5) **Population type:** Adolescents **Sample size:** 1473	Substance use (Prevention)	**Name:** SmartCoach **Type:** Mobile application; AI/ML **Description:** The intervention elements of the program were based on social cognitive theory. The key concepts were (1) outcome expectations, (2) self-efficacy, (3) observational learning, (4) facilitation, and (5) self-regulation.	**Quantitative:** Problem drinking and alcohol use in the preceding 30 days; Rate for smoking abstinence and the quantity of cigarettes smoked in the preceding 30 days; Cannabis use; Perceived stress; Well-being. **Qualitative:** -	In the 30 days preceding the 6-month follow-up assessment, prevalence of problem drinking increased by 2.5% in the intervention group and by 3.4% in the control group, relative to that observed at baseline. Quantity of alcohol consumed per month decreased by 0.6 standard drinks in the intervention group and increased by 0.7 standard drinks in the control group. The prevalence of tobacco

								smoking also showed a steeper increase for controls (from 15.1% to 18.5%) compared to those who received the intervention (from 12.1% to 14.5%) from baseline to the 6-month follow-up, but this effect was not significant. No significant group effect was observed for frequency of cannabis smoking. Pre-post differences in well-being and social skills did not differ significantly between groups.
Hides et al. (2019) [48]	Australia (schools)	RCT	To examine the effects of using Music eScape on emotion regulation, distress, and well-being at 1, 2, 3, and 6 months.	**Age (years):** 16-25 (Mean = 19.9) **Population type:** Young people **Sample size:** 169	Emotion dysregulation; distress; well-being (Prevention)	**Name:** Music eScape **Type:** Mobile application; AI/ML **Description:** The Music eScape app analyzes each song in the users' music library according to its level of valence and arousal using The EchoNest music data program. The songs are then located in a two-dimensional space consistent with Russell circumplex model of emotion, labelled around the borders with 8 emotions: aggressive, excited, happy, chilled, peaceful, bored, depressed, and stressed.	**Quantitative:** Emotion regulation, distress, well-being; Moderating effects; App use and quality. **Qualitative:** -	No significant differences between immediate and delayed groups on emotion regulation, distress, or well-being were found at 1 month. Both groups achieved significant improvements in 5 of the 6 emotion regulation skills, mental distress, and well-being at 2, 3, and 6 months. Unhealthy music use moderated improvements on 3 emotion regulation skills. High mean quality rating 3.8/5.

						Before creating a playlist, the app prompts users to reflect on their current and desired mood and then encourages them to plot a mood journey the playlist will support them to create. This journey comprises a unique trajectory using their own music.		
Holt-Quick et al. (2020) [65]	New Zealand (community)	AI/ML training/ validation	To describe the architecture underlying the chatbot.	**Age (years):** Not mentioned **Population type:** Youth **Sample size:** Not mentioned	Resilience (Promotion)	**Name:** Headstrong **Type:** Chatbot **Description:** Headstrong developed with methods grounded in CBT and positive psychology. The architecture supports a range of over 20 activities delivered in a 4-week program by relatable personas.	**Quantitative:** - **Qualitative:** -	Description of the architecture of the chatbot.
Huberty et al. (2019) [44]	USA (schools)	RCT	To test the initial efficacy and sustained effects of an 8-week mindfulness meditation mobile app compared to a wait-list control on stress, mindfulness, and self-compassion in college students with elevated stress.	**Age (years):** ≥18 (Mean = 20.41) **Population type:** Students **Sample size:** 88	Stress; mindfulness; self-compassion (Prevention)	**Name:** Calm **Type:** Mobile application **Description:** Calm is a consumer-based mindfulness meditation mobile app that offers a range of mindfulness meditation practice guide modules that vary in length, instruction, and content, and integrates some CBT techniques.	**Quantitative:** Stress, mindfulness, and self-compassion; Health behaviors; Feasibility and acceptability of the app. **Qualitative:** -	Significant differences in all outcomes after adjustment for covariates post-intervention. These effects persisted at follow-up, except for the non-reacting subscale of mindfulness. A significant group-time interaction in models of sleep disturbance was found, but no significant effects were found for other health behaviors. Majority reported that Calm was helpful to reduce stress and

								stated they would use Calm in the future. The majority were satisfied using Calm and likely to recommend it to other college students.
Kaess et al. (2019) [40]	Germany (schools)	RCT	To develop, implement, and evaluate an Internet-based program to promote help-seeking in children and adolescents with mental health problems across all disorders in a RCT.	**Age (years):** ≥12 (Mean = 20.41) **Population type:** Children and adolescents **Sample size:** 1500	General mental health problems (Promotion and prevention)	**Name:** ProHEAD online **Type:** Web interface **Description:** Receive online access to tailored information and individual advice on where to seek professional help for their specific needs close to their place of living, case reports of and interaction with peers, as well as the opportunity for online and telephone counselling.	**Quantitative:** Number of C&A seeking conventional face-to-face professional help in the real-world setting within 1 year after their initial screening; Help-seeking intentions, actual help-seeking behavior, and attitudes toward help-seeking; Health-related QoL. **Qualitative:** -	No results; protocol paper.
Kasson et al. (2021) [88]	USA (community)	Cohort	To leverage social media to connect with teens with eating disorders to identify population specific characteristics and to gather feedback on an mHealth intervention.	**Age (years):** 14-17 **Population type:** Teens **Sample size:** 30	Eating disorders (Promotion and prevention)	**Name:** Space from Body and Eating Concerns Program (SBEC) **Type:** Mobile application **Description:** Six modules based on CBT for EDs. Modules aim to promote a better understanding of how disordered eating behaviours develop and are maintained and to help users learn strategies for	**Quantitative:** Eating disorder symptoms; Depression; Anxiety. **Qualitative:** Feedback on app; Usability.	Participants from both groups experienced extremely high rates of depression and/or anxiety symptoms in addition to eating disorder symptoms. Positive impact of the app on eating disorder recovery. Useful in comparison to in-person treatment. Improvements can be made for app

						implementing regular eating, identifying and reframing ED-maintaining thoughts, managing feelings and emotions that may trigger ED behaviours, and using coping skills to reduce ED symptom severity.		content, presentation, and the inclusion of gamification or incentives for use.
Kenny et al. (2020) [56]	Ireland (schools)	RCT	To test the effectiveness of CopeSmart, a mental health mobile app which promotes self-management through emotional self-monitoring and the use of positive coping strategies.	**Age (years):** 15-18 (Mean = 16.05) **Population type:** Students **Sample size:** 560	Emotional distress; well-being; emotional self-awareness; coping (Prevention)	**Name:** CopeSmart **Type:** Mobile application **Description:** The app allowed them to rate how happy, angry, sad, stressed or worried they felt on a scale of 1-10 and users were able to review their ratings over time. The app provided a series of positive coping tips. A tip was randomly selected to display each time a user completed a mood rating and all tips were available for users to browse at their leisure.	**Quantitative:** Mental health symptoms. **Qualitative:** -	No significant changes in the intervention group from pre-test to post-test, when compared to the control group, in terms of emotional distress, well-being, emotional self-awareness or coping strategies.
Gillis G. (2015) [27]	Afghanistan (community)	Cohort	To design and test an intervention for strengthening a mental health system that improves awareness in the community, informs health practitioners, and makes	**Age (years):** 18-25 **Population type:** Young adults **Sample size:** 1200	Depression; drug addiction (Promotion)	**Name:** - **Type:** Foundational technology; mobile application **Description:** SMS messages are sent to young adults in the community to create awareness about MH problems. Smartphone	**Quantitative:** Feasibility. **Qualitative:** -	The mental health mobile application is in use by 95 CHWs and 25 facility-based health workers, serving a target population of over 100,000 in all four case districts. Over 10,000 text messages have been sent to 1200 registered adolescents. 51 sessions

			treatment accessible.			application for health providers to empower them to provide MH services in the community. Also integrated an eLearning platform and telehealth.		for the community and 33 blended learning sessions for health providers have been attended by 8,006 community members and 596 health providers.
Kuhn et al. (2017) [32]	Switzerland, Germany (community, clinics)	AI/ML training/ validation	To assess whether data should be collected from youth, parents, or both for effective mental health screening in adolescents.	**Age (years):** 11-17 (Mean = 13.98 Germany; 13.99 Switzerland) **Population type:** Adolescents **Sample size:** 252 (Germany); 86 (Switzerland)	General mental health (Prevention)	**Name:** DAWBA **Type:** Foundational technology; AI/ML **Description:** Structured interview sections covering the major mental disorders, followed by a semi-structured part eliciting open-ended descriptions about areas of concern. Diagnostic predictions, DAWBA bands, can be generated by computerized algorithms that combines information from symptom and impact measures from all available respondents.	**Quantitative:** Parent- and self-rated SDQ and DAWBA. **Qualitative:** -	Using ROC analyses and kappa statistics, both SDQ and DAWBA measures were suc- cessfully predicting the presence of an ICD-10 disorder as well as clinic sample status. Kappa statistics confirmed the hypothesis that there was an informant gradient: youth self- reports were less useful than parent reports for predicting diagnosis, whereas combined parent and youth reports were more discriminating.
Kutok et al. (2021) [71]	USA (community)	RCT	To evaluate the feasibility and acceptability of and obtain preliminary outcome data on IMPACT (Intervention Media to Prevent Adolescent Cyber-Conflict Through Technology), a brief, remote app-based intervention	**Age (years):** 13-17 (Mean = 15.3) **Population type:** Adolescents **Sample size:** 80	Cyberbullying (Prevention)	**Name:** IMPACT **Type:** Mobile application **Description:** The fully automated app delivered a daily query, at the time of the participants' choice. On the basis of the participants' responses as well as their baseline characteristics, the participants then received an automated, tailored intervention message	**Quantitative:** Cybervictimization; Bystander self-efficacy; Well-being; Feasibility; Acceptability. **Qualitative:** Message content and tone.	A significant treatment effect was observed on the number of solutions tried for combating personal cybervictimization, with intervention participants reporting a significantly higher number of strategies at 8 weeks. Participants in the intervention group attempted significantly more bystander interventions than those in

			to prevent and reduce the effect of cyberbullying.			based on MI, CBT, and bystander intervention methodologies.		the control group at 8 weeks. At both 8 and 16 weeks, well-being was significantly higher and psychological stress was lower among IMPACT users than among control group users. The retention rate was 99% at 8 weeks and 96% at 16 weeks for all participants. Daily response rate of 89%. 100% of the intervention participants were at least moderately satisfied with IMPACT overall, and 92% of the participants were at least moderately satisfied with the app.
Lee et al. (2018) [33]	Canada (schools)	RCT	To evaluate a mindfulness-based app' efficacy on stress, anxiety, depressive symptomology, sleep behaviour, work or class absenteeism, work or school productivity, and quality of life (QoL) among university students.	**Age (years):** Mean = 20.3 **Population type:** Students **Sample size:** 163	Stress; anxiety; depressive symptomatology; sleep quality; health-related QoL (Prevention)	**Name:** DeStressify **Type:** Mobile application **Description:** The app contains a core plan that delivers mindfulness-based exercises through audio, video, or text files. Example titles of these exercises include grounding visualization, gratitude, imagining the life you want, and finding meaning.	**Quantitative:** Mental health symptoms; Productivity. **Qualitative:** -	Reduce trait anxiety and improve general health, energy, and emotional well-being. The app did not significantly improve stress, state anxiety, physical and social functioning, and role limitations because of physical or emotional health problems or pain. More participants in the experimental condition believed their productivity improved between baseline and post-intervention measurements than the number of participants

								expected to believe so randomly by chance.
Lee et al. (2021) [69]	Australia (schools)	Economic evaluation	To analyse whether an online e-health intervention to prevent anxiety disorders among school students represents good value for money.	**Age (years):** 11-17 **Population type:** Students **Sample size:** Not mentioned	Anxiety (Prevention)	**Name:** MoodGYM **Type:** Web interface **Description:** Five online modules based on CBT, consisting of written information, animations, interactive exercises and quizzes.	**Quantitative:** Anxiety symptoms; Cost-effectiveness. **Qualitative:** -	MoodGYM intervention reduced the risk of developing anxiety by 21% immediately after receiving the intervention and 36% around 6 months after receiving the intervention. MoodGYM intervention was observed to produce net cost savings while simultaneously producing positive QALY health gains.
Manicavasagar et al. (2014) [25]	Australia (community)	RCT	To explore the feasibility of the online delivery of a youth positive psychology program, Bite Back, to improve the well-being and mental health outcomes of Australian youth.	**Age (years):** 12-18 (Mean = 15.4) **Population type:** Youth **Sample size:** 154	Depression ; anxiety; stress; well-being (Promotion and prevention)	**Name:** Bite Back **Type:** Web interface **Description:** Positive psychology exercises and information. A range of interactive activities including making gratitude entries, mindfulness meditations, describing personal stories, and a mindfulness exercise involving taking photos.	**Quantitative:** Depression, anxiety, stress, well-being. **Qualitative:** Acceptability; Reasons for under-usage.	Participants in the Bite Back group with high levels of adherence reported significant decreases in depression and stress and improvements in well-being. Bite Back users who visited the site more frequently reported significant decreases in depression and anxiety and improvements in well-being. No significant improvements were found among Bite Back users who demonstrated low levels of adherence or who used the website less frequently. 79% of Bite Back users reported positive experiences using the website. Reasons for

								under-usage included time constraints, technical issues, relevance and website content.
Moltrecht et al. (2021) [73]	UK (schools)	Cohort	To optimize the usability, acceptability, and utility of the new app-based intervention designed to support children' emotion regulation in schools, and explore its scope for implementation with the target user in the school context.	**Age (years):** 10-12 (Mean = 10.5) **Population type:** Youth **Sample size:** 144	Depressive symptoms; well-being; emotion regulation (Prevention)	**Name:** - **Type:** Mobile application **Description:** Four modules: play, relax (mindfulness and relaxation exercises), watch (psychoeducational animations), and tools (emotion regulation strategies), which provide users with the opportunity to learn, practice, and develop new emotion regulation skills.	**Quantitative:** Acceptability; Usability. **Qualitative:** Acceptability; Barriers to implementation.	30% were returning users. 67% reported the app was helpful. Most features functioned well; however, certain technical issues were reported, which may have led to reduced engagement levels. Participants reported that the app helped them to calm down and relax. They highlighted the need for new, activating app features in addition to the existing, primarily relaxing ones.
Nagamitsu et al. (2022) [95]	Japan (schools)	RCT	To test the efficacy of two adolescent health promotion interventions: a well-care visit (WCV) with a risk assessment interview and counselling and self-monitoring with a smartphone CBT app.	**Age (years):** 13-18 **Population type:** Adolescents **Sample size:** 128	General mental health (Promotion and prevention)	**Name:** Mugimaru (CBT app) **Type:** Mobile application **Description:** Psychoeducation session and self-monitoring session. Psychoeducation was presented in a story-like manner, so that the adolescents can easily understand the rationale of CBT and were motivated to continue using the app.	**Quantitative:** Depression self-rating scale for children; Adolescent Health Promotion Short Form for health-promoting behaviors; Self-esteem scale and health-related quality of life; Suicidal ideation. **Qualitative:** -	A significant temporary effect of improving depressive symptoms at the onset of the intervention in high school students irrespective of intervention type. Significant increase in health promotion scale scores at the 4-month follow-up in junior high school students in the WCV group. No significant difference for self-esteem scale and health-related quality of life. Significant effect in terms of reduced

								suicidal ideation in adolescents during the 4-month observation period in both the WCV group and the WCV with CBT app group.
Neal-Barnett et al. (2019) [50]	USA (schools)	Cohort	To evaluate the effectiveness of the BYOTS app.	**Age (years):** 12-15 **Population type:** Adolescent females **Sample size:** 72	Negative thinking; anxiety (Prevention)	**Name:** Build Your Own Theme Song (BYOTS) **Type:** Mobile application; AI/ML **Description:** The BYOTS mHealth app is integrated into an eight-session culturally infused Sisters United Now (SUN) sister circle that takes place during the school day. Consists of a self-penned, self-recorded theme song based on the girl's favourite song. Girls are introduced to the negative and positive thought cycles. They learn how a theme song can interrupt the negative thought cycle by replacing the negative thoughts with positive affirming thoughts.	**Quantitative:** Negative thinking; Anxiety. **Qualitative:** Effectiveness.	Negative thinking was significantly lower at day 7 than day 1. Anxiety from preuse to postuse of the app was also reduced. Participants found the app promoted calmness, and it is helpful in stressful home situations.
Nguyen-Feng et al. (2017) [28]	USA (schools)	RCT	To evaluate the efficacy of Internet-based stress management programs for college students.	**Age (years):** 18-21 **Population type:** Students **Sample size:** 365	Stress coping; depression; anxiety (Prevention)	**Name:** - **Type:** Web interface **Description:** First watched a mindfulness psychoeducational video. Over the weeks, participants listened to a downloadable guided	**Quantitative:** Self-reported measures of stress, anxiety, depression; IPT as moderator. **Qualitative:** -	All 3 groups reported significant decreases on all primary outcomes, and time-by-intervention group interaction effects suggest that the 3 conditions were equally effective. When examining IPT history as a

						meditation focusing on 1) the breath, 2) noticing and attending to physical sensations, 3) attending nonjudgmentally to thoughts and feelings that arose during practice, 4) cognitions and sensations. They also completed mindfulness logs in which they briefly described their experience and reported any difficulties encountered.		moderator, the mindfulness plus present control and stress management conditions were both more effective for IPT survivors than the mindfulness only intervention.
O'Dea et al. (2020) [52]	Australia (community)	RCT	To evaluate the acceptability and effectiveness of a relationship-focused mobile phone application (WeClick) for improving depressive symptoms and other mental health outcomes in adolescents.	**Age (years):** 12-16 (Mean = 14.82) **Population type:** Adolescents **Sample size:** 193	Depression; anxiety; psychological distress; well-being; help-seeking (Promotion and prevention)	**Name:** WeClick **Type:** Mobile application; AI/ML **Description:** Interactive story-telling app that consists of four characters, each facing different relationship difficulties and other adolescent issues. A series of activities to develop skills to overcome negative thinking and problem solving.	**Quantitative:** Depressive symptoms; Wellbeing, help-seeking intentions and professional help-seeking intentions for mental health problems; Generalised anxiety and separation anxiety, social self-efficacy or any social support outcomes; Acceptability. **Qualitative:** -	Change in depressive symptoms from baseline to 4-week post-test did not differ significantly between intervention and control. Increases in help-seeking intentions were sustained at follow-up in the intervention condition. No differential effects were found for anxiety or social support outcomes. Over 90% of participants indicated the app was enjoyable, interesting and easy to use.
Ospina-Pinillos et al.	Colombia (community)	Qualitative	To conduct co-design workshops to culturally adapt	**Age (years):** 16-30 (Median =	Well-being (Prevention)	**Name:** MHeC-C **Type:** Web interface	**Quantitative:** -	Participants liked the idea of having an MHeC designed and adapted for

(2020) [53]			MHeC for young people in Colombian, and perform one-on-one user-testing sessions to evaluate an alpha prototype of MHeC-C.	19.5 for co-design workshops; 22 for user testing) **Population type:** Young people **Sample size:** 18 for co-design workshops, 10 for user testing		**Description:** 6 iterative phases: co-design workshops; knowledge translation; tailoring to language, culture, and place (or context); and one-on-one user-testing sessions (alpha, beta, delta prototype).	**Qualitative:** Opinions about the MHeC-C.	Colombian young people. It is important for MHeC-C to be embedded in the public health care system. 5 key elements were acceptable (home page and triage system, self-report assessment, dashboard of results, booking and video-visit system, and personalized well-being plan). Stressed the need to develop additional functionality (phone network backup; chat; geolocation; and integration with electronic medical records, apps, or electronic tools).
Parada et al. (2020) [55]	Chile (schools)	Qualitative	To evaluate Cuida tu Animo, an internet-based pilot program for prevention and early intervention of adolescent depression via persuasive systems design (PSD).	**Age (years):** Not mentioned **Population type:** Adolescents **Sample size:** Not mentioned	Depression (Prevention)	**Name:** Cuida tu Animo **Type:** Web interface **Description:** Adolescents interacted with the program via monitoring and feedback messages, which were delivered every 2 weeks, and a website that allowed them to access psychoeducational content, provided emergency information and allowed users to contact a specialist via online chat appointments.	**Quantitative:** - **Qualitative:** Evaluated for PSD features (primary task support, dialogue support, social support, system credibility support).	CTA provided a pleasant visual environment for the adolescents to receive the intervention, as well as means to complete assessments and interact with the website content, but was more ineffective at proposing specific easy-to-accomplish tasks for its users and did not allow participants to interact with each other in any way.

Pine et al. (2021) [83]	New Zealand (community)	Technology design & development	To present the development of a Casual Video Game to promote psychological well-being among adolescents aged 13 to 15 years.	**Age (years):** 13-15 **Population type:** Adolescents **Sample size:** Not mentioned	Well-being (Promotion)	**Name:** Match Emoji **Type:** Mobile application; AI/ML **Description:** The goal of Match Emoji is to match similar coloured emojis together to earn points and progress through the game. The six different coloured and shaped emojis in the game represent digital expressions of emotions, ideas, and personality. Each micro-message in Match Emoji is assigned to one of nine different message sets to support the learning of psycho-logical well-being skills.	**Quantitative:** - **Qualitative:** -	Description of the design of Match Emoji.
Punukollu et al. (2020) [62]	UK (schools)	Qualitative	To present qualitative results for the benefits of the app SafeSpot.	**Age (years):** 11-15 **Population type:** Youth **Sample size:** 650	General mental health (Promotion)	**Name:** SafeSpot **Type:** Mobile application; web interface **Description:** It combines classroom tutorials on mental well-being, a website and a mobile app, to develop coping mechanisms. It also includes a programme through which older pupils, known as SafeSpotters, are trained in SafeSpot material to provide guidance to younger pupils to improve their mental health and reduce stigma.	**Quantitative:** - **Qualitative:** Pupil engagement; Content of tutorials; Perceived impact of SafeSpot.	Some found that the app was a useful way to enhance the content, others did not show the same level of interaction with the app. The content of tutorials may need to be altered. The lessons could be more interactive to increase the pupils' participation and enjoyment. Positive impact of SafeSpot: improved understanding and normalisation of mental health problems.

Rameza nian et al. (2019) [51]	Finland (community)	AI/ML training/ validation	To discuss how to detect cyberbullying with AI techniques in a privacy-preserving way.	**Age (years):** Not mentioned **Population type:** General population **Sample size:** Not mentioned	Cyberbullying (Prevention)	**Name:** - **Type:** AI/ML **Description:** Detect a text message that contains cyberbullying keywords in a privacy-preserving way, without revealing the context of a benign message to the operator. Uses AI methods to classify the message as bully or benign. If the message is benign, it goes through and otherwise it will be blocked.	**Quantitative:** - **Qualitative:** -	Description of the design of the AI system.
Ranney et al. (2021) [70]	USA (hospitals)	Cohort	To gather, analyse and use qualitative data in real time for iterative intervention refinement of a mixed-modality cyberbullying intervention.	**Age (years):** 13-17 (Mean = 15) **Population type:** Youth **Sample size:** 21	Cyberbullying (Prevention)	**Name:** Intervention to Prevent Adolescent Cybervictimization with Text message (iPACT) **Type:** Mobile application **Description:** An 8-week, daily, automated, two-way text messaging curriculum. Participants could pull additional message content by texting keywords. Content was based on in-person and SMS text message-based violence prevention and cyberbullying prevention interventions.	**Quantitative:** Feasibility; Acceptability. **Qualitative:** Feedback on intervention.	Of the 1064 daily queries sent to participants across all three iterations, 88.34% received a response. Participants responded to both questions on 73.59% of days. Average rate of response for both questions was 61% for session 1, 83% for session 2 and 81% for session 3. Overall quality rated as good or excellent.
Schaefer et al. (2021) [87]	USA (hospitals)	Descriptive	To examine the transition of BART interventions to a virtual format.	**Age (years):** Not mentioned **Population type:** Youth	Stress (Prevention)	**Name:** BART **Type:** Emerging technology; AI/ML **Description:** Using screen-sharing and an annotation	**Quantitative:** - **Qualitative:** -	Protocol changes illustrated the need for more thorough description of treatment sessions, increased focus on privacy and ethical

				**Sample size:** Not mentioned		capability on a telehealth platform helped build collaboration in identifying the different types of stressors in the patient's life. Through the use of shared documents and fill-in-the-blank exercises, clinicians could demonstrate types of stressors while seeking input from patients, thus actively implementing the CBT approach.		considerations, and age-related factors.
Schueller et al. (2019) [42]	USA (community)	RCT	To deploy and evaluate a mobile phone-based intervention to address the mental health needs of young adults experiencing homelessness.	**Age (years):** 18-24 (Mean = 19.34) **Population type:** Young adults **Sample size:** 35	Depression; post-traumatic stress disorder; emotion regulation (Prevention)	**Name:** Pocket Helper, IntelliCare **Type:** Mobile application **Description:** Pocket Helper is an app that consists of a daily survey and a daily coping skills-focused tip. Data entered into the app were displayed as feedback to the participant and sent to a coach dashboard to allow the coach to incorporate this information into their phone sessions. IntelliCare is a modular treatment suite consisting of 13mini-apps, each focused on a singular behaviour change technique drawn from CBT and positive psychology.	**Quantitative:** Feasibility; Acceptability; Depression; Post-traumatic stress disorder; Emotion regulation. **Qualitative:** -	57% completed all 3 of their phone sessions. Higher rates of satisfaction among the participants with valid responses. Very little change from pre- to post-treatment on measures of depression (*d*=0.27), post-traumatic stress disorder (*d*=0.17), and emotion regulation (*d*=0.10).

Serlachius et al. (2021) [72]	New Zealand (community)	Cohort	To explore the acceptability, usability, and preliminary efficacy of Whitu before refining the app for a RCT.	**Age (years):** 16-25 (Mean = 21) **Population type:** Young people **Sample size:** 20	Well-being; depression; anxiety; stress; self-compassion (Prevention)	**Name:** Whitu **Type:** Mobile application **Description:** Well-being app with 7 modules (Feel; Relax; Be kind to yourself; Be thankful; Connect; Look after your body; Set goals) that support young people to learn and practice evidence-based coping skills, including relaxation, mindfulness, self-compassion, and goal-setting. Each module contains at least one informative video that provides a graphical and audio description of its content.	**Quantitative:** Anxiety, stress, well-being, depression; Self-compassion, optimism, sleep quality. **Qualitative:** Factors affecting engagement; Issues with functionality; Preferences regarding aesthetics; Effectiveness and adverse effects.	Statistically significant improvements in anxiety and stress from baseline to 2-weeks post intervention. Improvements in well-being, depression, anxiety, and stress were also observed from baseline to 6-weeks. No statistically significant changes to self-compassion, optimism, sleep quality.
Shaukat-Jali et al. (2021) [86]	UK (community)	AI/ML training/ validation	To investigate whether subclinical social anxiety in young adults can be detected using physiological data obtained from wearable sensors, including heart rate, skin temperature, and electrodermal activity (EDA).	**Age (years):** 18-23 (Mean = 19.75) **Population type:** Young adults **Sample size:** 12	Social anxiety (Prevention)	**Name:** - **Type:** Emerging technology **Description:** Physiological data (heart rate, skin temperature, and electrodermal activity) were collected using an E4 Empatica wearable device. Using the preprocessed data and following a supervised machine learning approach, various classification algorithms were used to develop models for 3 different contexts. Models were trained to differentiate	**Quantitative:** Accuracy; Modalities. **Qualitative:** -	With modalities combined, the developed models yielded accuracies between 97.54% and 99.48% when differentiating between baseline and socially anxious states. Models trained to differentiate among baseline, anticipation anxiety, and reactive anxiety states yielded accuracies between 95.18% and 98.10%. Models developed to differentiate between social anxiety experienced by individuals with anxiety of

						social anxiety of differing severity.		differing severity scores successfully classified with accuracies between 98.86% and 99.52%. EDA was identified as the most effective singular modality when differentiating between baseline and social anxiety states, whereas skin temperature was the most effective modality when differentiating anxiety among individuals with social anxiety of differing severity.
Sia et al. (2021) [89]	Philippines (schools)	Cohort	To describe the design and evaluation of Abot to encourage students to improve their lifestyle habits and well-being.	**Age (years):** 17-18 **Population type:** Students **Sample size:** 25	Well-being (Promotion and prevention)	**Name:** Abot **Type:** Chatbot **Description:** Chatbot deployed in Facebook Messenger. 1. Assessment (healthy or unhealthy lifestyle): discusses a specific aspect of well-being or lifestyle habit through story sharing. 2. Counselling: advice and solutions in changing lifestyle habits given. 3. Evaluation: user to give feedback on the usefulness of the conversation in encouraging him/her to practice better lifestyle habits.	**Quantitative:** Well-being; Perceived user acceptability of chatbot. **Qualitative:** -	Positive improvement in their physical well-being, positive change in their social well-being, and positive change in their academic well-being. User acceptability: Out of 4, an average score of 3.35 on performance, 3.36 on humanity, and 3.68 on affect.

Srivastava et al. (2017) [29]	India (community)	Technology design & development	To develop an electronic platform for e-psychology systems to deal with various health issues of adolescents.	**Age (years):** 10-19 **Population type:** Adolescents and young adults **Sample size:** Not mentioned	Psychological distress (Prevention)	**Name:** Yuva **Type:** Web interface **Description:** Electronic platform for e-psychology systems by using ICT tools and services and can be used to deal with various health issues of adolescents. It incorporates several facilities like self-assessment tests, registration facility for counsellors, dieticians, instructors etc. so that they can be contacted at the hour of need.	**Quantitative:** - **Qualitative:** -	Description of the design of the Yuva platform.
Srividya et al. (2018) [37]	India (community)	AI/ML training/ validation	To apply ML algorithms to identify state of mental health in a target group.	**Age (years):** 18-21 **Population type:** Students **Sample size:** 656	Psychological distress; Well-being (Prevention)	**Name:** - **Type:** AI/ML **Description:** Questionnaire looks at 5 factors: engagement, perseverance, optimism, connectedness, and happiness. Based on scores obtained, a person's MH status is predicted as mentally distressed, barely satisfied with life, and optimistic.	**Quantitative:** Accuracy; Precision. **Qualitative:** -	SVM, KNN, ensemble (bagging) and tree ensemble (random forest) give an equivalent accuracy score of 0.9. SVM, KNN, ensemble (bagging) and tree ensemble (random forest) give a score of 0.95 for the mentally distressed class label.
Stephens et al. (2020) [66]	USA (community)	RCT	To summarize the process of recruiting and engaging American Indian and Alaska Native teens and young adults in a	**Age (years):** 15-24 **Population type:** Teens and young adults	Help-seeking (Promotion)	**Name:** BRAVE **Type:** Mobile application **Description:** The BRAVE campaign included 3-5 text messages per week, including 1 role model video per week and a	**Quantitative:** Response rate. **Qualitative:** -	During recruitment, Facebook Ads with a positive tone had higher visibility than purely descriptive ads. Text messages with a call to action had higher response

			culturally relevant behavioural health intervention in the U.S.	**Sample size:** 1030		related image. The intervention was designed to amplify and reinforce healthy social norms and cultural values, teach suicide warning signs, prepare youth to initiate difficult conversations with peers and trusted adults, encourage youth to access mental health resources, destigmatize mental health services and connect youth to trusted adults.		rates, suggesting that BRAVE text messages should be interactive and prompt users to engage rather than just report back.
Stephens et al. (2021) [90]	USA (schools)	Cohort	To assess feasibility and usability of an online Teen Wellness (TW) platform designed to teach cognitive behavioural coping and resiliency skills.	**Age (years):** 13-14 (Mean = 13.125) **Population type:** Students **Sample size:** 16	Well-being; Resilience (Prevention)	**Name:** Teen Wellness (TW) **Type:** Web interface **Description:** Interactive, online, wellness education program, which provides resiliency skills using specific CBT. 13 modules that focused on different resiliency skills, which were accompanied by a brief video.	**Quantitative:** Usefulness; Life satisfaction. **Qualitative:** -	Rated 7.62 out of 10 for usefulness. 68.8% of participants completed all modules. Significant changes in satisfaction with 'friendships' and 'self'. No significant changes on other measures.
Stiles-Shields et al. (2022) [91]	USA (community)	Cohort	To describe the development and feasibility evaluation of the Emotional Needs Evaluation and Resource Guide for You (ENERGY) System, a DMH tool to meet the	**Age (years):** Mean = 18.95 **Population type:** Adolescents and young adults **Sample size:** 75	Depression; anxiety; trauma; alcohol and substance use (Prevention)	**Name:** Emotional Needs Evaluation and Resource Guide for You (ENERGY) System **Type:** AI/ML **Description:** The ENERGY System offers a brief assessment of resource needs; problem-solving capabilities; and symptoms	**Quantitative:** Mental health intervention domains; Satisfaction; Resource needs. **Qualitative:** -	Triggered on average 2.04 intervention domains. Behavioral Activation/Increasing Activities was the most frequently launched intervention domain, and domains related to alcohol or substance use were the least frequent. The most

			mental health and resource needs of youth and their families developed in the context of the COVID-19 pandemic.			of depression, anxiety, trauma, and alcohol and substance use followed by automated, personalized list of appropriate self-help and other resources.		frequently requested support areas were finances, transportation, and food. Participants were satisfied with the ENERGY System overall as well as the length of time it took to answer the questions, which they found easy to answer. Half who completed resource needs questions indicated that they did not need assistance with any resources. The average number of resource categories provided to participants was 0.69.
Stoyanov et al. (2021) [79]	Australia (community)	Qualitative	To explore how young Australians conceptualize and construct recovery journeys from feeling unwell to being well in order to inform the conceptual design of a youth-led information-, resource-, and support-focused mHealth intervention.	**Age (years):** 12-25 **Population type:** Young people **Sample size:** 25	Well-being (Prevention)	**Name:** The Niggle App **Type:** Mobile application; AI/ML **Description:** Through co-design, the new app was conceptualized as a visual representation of an amorphous problem, which can be addressed through app functions corresponding to the most helpful strategies that young people used to progress through the stages of their recovery journey.	**Quantitative:** - **Qualitative:** 6-stage journey with specific barriers and coping strategies.	Description of the design of The Niggle App.

Trappey et al. (2022) [100]	Taiwan (any)	RCT	To develop an empathy-centric counselling chatbot system that is capable of sentimental dialogue analysis that can help students allay their distress and psychological problems.	**Age (years):** 20-26 (Mean = 22.77) **Population type:** Students **Sample size:** 178	Stress (Prevention)	**Name:** VRECC (VR empathy-centric counselling chatbot) **Type:** Chatbot; emerging technology **Description:** Students can use VR devices to connect to the system. The system consists of three components, including a questionnaire to measure users' stress status, a chatbot to talk with users, and a chat room where users can interact with each other and talk freely with counsellors.	**Quantitative:** Stress level; Psychological sensitivity; Life impact. **Qualitative:** -	Significant reduction in stress level. Reduced psychological sensitivity. No significant result for life impact.
Uddin et al. (2022) [104]	Norway (community)	AI/ML training/ validation	To describe an approach to detect depression from text messages using Long Short-Term Memory-Based Neural Structured Learning.	**Age (years):** Not mentioned **Population type:** Youth **Sample size:** Not mentioned	Depression (Prevention)	**Name:** - **Type:** AI/ML **Description:** A text dataset is collected from the queries submitted in a Norwegian youth forum and then efficient features are obtained based on pre-defined handcrafted robust features developed by focusing on the symptoms of depression that were defined by medical practitioners and psychologists. After that, LSTM-based NSL approach is applied as deep learning method to train the features	**Quantitative:** Accuracy. **Qualitative:** -	Superiority against the traditional approaches on the dataset consists of Norwegian text, achieving the mean accuracy of 99% whereas the conventional approaches achieved a maximum of 96% mean accuracy.

						discriminating depression and non-depression. The trained LSTM-based NSL is then utilized later to detect depression in the testing text messages.		
Vigil-Hayes et al. (2021) [81]	USA (community)	Qualitative	To co-design a behavioral mHealth intervention with Native American youth.	**Age (years):** 12-18 **Population type:** Native youth **Sample size:** 11	Mindfulness; stress; resilience (Prevention)	**Name:** ARORA (Amplifying Resilience Over Restricted Internet Access) **Type:** Mobile application; AI/ML **Description:** mHealth intervention with evidence-based practices for increasing mindfulness with Hopi and Navajo visual themes. Four main types of positive psychological intervention activities: mindfulness, emotional and cognitive coping skills, cultivation of resiliency factors, and prosocial skills development.	**Quantitative:** - **Qualitative:** Co-design features.	Participants desired a greater level of didactic interaction with cultural and behavioral health elements, as well as explicit explanation of cultural significance, and more engagement with nature symbology. Immersiveness of the app activities, and customization of app are important for long-term sustainability.
Vorobyov et al. (2021) [67]	Russia (schools)	Technology design & development	To describe a digital health passport for monitoring physical, mental health and physical preparedness of the educational process participants in modern educational environment.	**Age (years):** 6-17 **Population type:** Students **Sample size:** Not mentioned	Well-being (Prevention)	**Name:** Health Passport **Type:** Mobile application; AI/ML **Description:** Objective evaluation of mental health through psychological methods such as The Ladder Test and The Self-Esteem Test. The programme can also give individualized practical recommendations by	**Quantitative:** Physical health; Mental health; Physical preparedness. **Qualitative:** -	Description of the design of the Health Passport platform.

						analyzing test results; store statistically relevant information on individual and group performance; conduct comparative analysis.		
Werner-Seidler et al. (2019) [46]	Australia (community)	Cohort	To test the feasibility, acceptability and preliminary effects of a recently developed smartphone application, Sleep Ninja, for adolescent sleep difficulties.	**Age (years):** 12-16 (Mean = 13.71) **Population type:** Young people **Sample size:** 50	Sleep difficulties; depression; anxiety (Prevention)	**Name:** Sleep Ninja **Type:** Mobile application; AI/ML **Description:** The core strategies covered by the app are psychoeducation, stimulus control, sleep hygiene and sleep-related cognitive therapy. It includes six training sessions, a sleep tracking function, recommended bedtimes based on sleep guidelines, reminders to start a wind-down routine each night, a series of sleep tips and general information about sleep.	**Quantitative:** Sleep variables; Feasibility. **Qualitative:** Acceptability.	Significant improvements on sleep variables including insomnia, sleep quality, depression and anxiety. 82% completed the first lesson, 51% completed four of the six lessons and 33% completed all six. Participants expressed a desire for improved explanation of the different app sections and what they needed to do each time they opened the app, as well as to be able to personalize their user experience more.
Werner-Seidler et al. (2020) [57]	Australia (schools)	RCT	To investigate whether cognitive behaviour therapies (CBT) delivered by smartphone application can prevent depression.	**Age (years):** 12-14 **Population type:** Students **Sample size:** 10000	Depression (Prevention)	**Name:** SPARX; Sleep Ninja **Type:** Mobile application **Description:** SPARX-Future Proofing: gaming intervention. Instead of a focus on existing symptoms and depression, terminology has been updated to focus on prevention, and times when the participants have felt	**Quantitative:** Symptoms of depression, psychological distress, anxiety, insomnia. **Qualitative:** -	No results; protocol paper.

						down or stressed, and included skills and strategies reframed to focus on dealing with problems as they arise. SPARX consists of seven modules which cover: finding hope, being active, dealing with strong emotions, problem-solving, recognising unhelpful thoughts, challenging unhelpful thoughts and bringing it all together.		
Wiljer et al. (2020) [63]	Canada (schools)	RCT	To examine the impact of Thought Spot on mental health and wellness help-seeking intentions, behaviours, attitudes, self-stigma, and self-efficacy among postsecondary students in Canada.	**Age (years):** 17-29 **Population type:** Students **Sample size:** 481	Well-being; help-seeking (Promotion and prevention)	**Name:** Thought Spot **Type:** Mobile application; web interface **Description:** Standalone app that allows users to add, review, and search crowdsourced information about nearby mental health and wellness services.	**Quantitative:** Formal help-seeking intentions; Informal help-seeking intentions and help-seeking behaviors, help-seeking attitudes, self-stigma, and self-efficacy. **Qualitative:** Usability of Thought Spot.	Both groups demonstrated similar increases in formal help-seeking intentions at 3 and 6 months. Females were less likely to seek help from informal sources than males. Most participants stopped using the platform after 3 weeks. Participants searched and were interested in a variety of resources, including mental health, counselling and social services.
Woodrow et al. (2022) [97]	UK (schools)	Qualitative	To evaluate the perspectives and experiences of young people who have taken part in a novel school-based health and wellbeing screening	**Age (years):** 13-14 **Population type:** Young people **Sample size:** 51	Well-being (Prevention)	**Name:** Digital Health Contact (DHC) **Type:** Foundational technology **Description:** Online health and wellbeing questionnaire (universal screening tool) delivered to	**Quantitative:** - **Qualitative:** Perceived acceptability of the DHC; Delivery mode.	Participants indicated high acceptability of DHC as an useful approach for identifying health needs and providing support. They also appreciated the online and home-based approach (more private,

			programme, the Digital Health Contact (DHC), which has linked follow-up support.			an entire school year group. The questions cover a range of physical and mental health topics. All young people are provided with a digital personalised care plan upon completion of the questionnaire, which contains generic public health advice and signposting to relevant support.		facilitated more honest responses than face-to-face, less time-pressured). A barrier to openness for young people was concern around who would be able to see their responses.
Woolderink et al. (2010) [24]	Netherlands (community)	RCT	To examine the (cost-) effectiveness of the Kopstoring intervention.	**Age (years):** 16-25 **Population type:** Youth **Sample size:** 124	Resilience; coping (Prevention)	**Name:** Kopstoring **Type:** Web interface **Description:** 8 consecutive online weekly chat group meetings and a ninth evaluation meeting in a chat box at the website. The aim of the intervention is to strengthen protective factors, such as self-management skills and psychological well-being, and prevent the development of psychological disorders. The participants are required to carry out homework assignments before entering the chat box meeting.	**Quantitative:** Internalizing and externalizing symptoms; Healthcare costs; Costs outside the healthcare sector. **Qualitative:** -	No results; protocol paper.
Yip et al.	Hong Kong (community)	Cohort	To discuss the structure of the operation, usage	**Age (years):** 11-35	General mental health	**Name:** Open Up **Type:** Web interface; AI/ML	**Quantitative:** Service indicators; User	Number of valid sessions increased gradually, and about 34.5% of the users

(2021) [78]			pattern and its effectiveness, the use of AI to improve users experience, and the role of volunteer in the operation.	**Population type:** Young adults **Sample size:** 29400	(Prevention)	**Description:** Enables people to chat with social workers or trained volunteers anonymously. It can be accessed anywhere and anytime for free through various channels, including SMS, WhatsApp, Facebook Messenger, and the official web portal. Six Smart Modules that, employing AI and NLP technologies, provides (i) real-time topic classification, (ii) risk assessment, (iii) practices reminders, (iv) recommendation for referrals or standardized responses (v) user segmentation, and (vi) big data analytics platform.	satisfaction; De-escalation of risk; Topics of concern. **Qualitative:** -	sought Open Up services multiple times. 81.5% of users found the service helpful and 85.4% were more likely to seek help in the future. Open Up lowered 85.3% of the high risk and crisis cases. Topics of concern included mental health/ emotion issues, family, intimate relationship, interpersonal relationship, study, medical issue, career prospects, and work pressure.
Zarglayoun et al. (2022) [99]	Canada (community)	RCT	To provide preliminary information on the development of a novel serious video game for assessing and optimizing socio-moral reasoning (SMR) in adolescence.	**Age (years):** 12-17 (Mean = 14.39) **Population type:** Adolescents **Sample size:** 57	Socio-moral reasoning (Prevention)	**Name:** MorALERT **Type:** Web interface; AI/ML **Description:** The game is played on a standard desktop computer and is in the third person perspective. Evaluative version: composed of nine everyday socio-moral dilemmas presented in a predetermined, continuous sequence. As the player nears a character, they provide their perspective on	**Quantitative:** Intellectual functioning; Socio-moral reasoning; Interpersonal reactivity index; Empathy; Sense of presence. **Qualitative:** -	Adolescents significantly improved their SMR by playing the game. No correlation was found between SMR, empathy and the sense of presence.

						the socio-moral dilemma presented. Adaptive version: relies on an automated coding algorithm based on NLP, deep learning and expert knowledge from which an immediate SMR maturity score is produced as players express their moral justifications.		
